# Click Inspired Synthesis of Novel Cinchonidine Glycoconjugates as Promising Plasmepsin Inhibitors

**DOI:** 10.1038/s41598-020-59477-3

**Published:** 2020-02-27

**Authors:** Nidhi Mishra, Anand K. Agrahari, Priyanka Bose, Sumit K. Singh, Anoop S. Singh, Vinod K. Tiwari

**Affiliations:** Department of Chemistry, Institute of Science, Banaras Hindu University, Varanasi, 221005 India

**Keywords:** Combinatorial libraries, Combinatorial libraries, Carbohydrate chemistry, Carbohydrate chemistry

## Abstract

Among all the malaria parasites, *P. falciparum* is the most predominant species which has developed drug resistance against most of the commercial anti-malarial drugs. Thus, finding a new molecule for the inhibition of enzymes of *P. falciparum* is the pharmacological challenge in present era. Herein, ten novel molecules have been designed with an amalgamation of cinchonidine, carbohydrate moiety and triazole ring by utilizing copper-catalyzed click reaction of cinchonidine-derived azide and clickable glycosyl alkynes. The molecular docking of developed molecules showed promising results for plasmepsin inhibition in the form of effective binding with target proteins.

## Introduction

Malaria has been a medical challenge for centuries mostly in tropical and subtropical countries of the world. Despite significant medical advancement in treatment and prevention of malaria, it still causes thousands of deaths every year^1^. The main cause of the disease is protozoan parasite *Plasmodium* which is generally transmitted by an infected female *Anopheles* mosquito. Out of five species of *Plasmodium* responsible for malaria in human, i.e. *Plasmodium falciparum*, *Plasmodium vivax*, *Plasmodium knowlesi, Plasmodium malariae* and *Plasmodium ovale*, *Plasmodium falciparum* is the most virulent and accounts most of the deaths caused by malaria. There are several anti-malarial drugs available in markets which act against malaria parasite with different modes of action, but due to rapid spread of drug resistance in malaria parasites mainly *Plasmodium falciparum*, the need of new antimalarial drug leads is increasing. Plasmepsins, enzymes produced by *Plasmodium falciparum*, play a key role in hemoglobin-degrading activity of the parasite by export of *Plasmodium* proteins to the host cell surface^2^. Plasmepsin I and II are known to play crucial role in hemoglobin catabolism by cleaving the hemoglobin alpha chain between Phe33 and Leu34 which are located in hinge region which results into dislocation and partial unfolding of globin subunits which further causes more protease sites within the globin polypeptide chains. At this stage, further degradation of large globin fragments takes place by action of plasmepsins and falcipains. In this regards, plasmepsins, which play a key role in the survival of *P. falciparum* in the host, have emerged as the new effective targets for development of antimalarial drugs with plasmepsin inhibition mode of action. Because of their key functioning in malaria symptoms and consequences, these enzymes are the main target of the anti-malarial drugs^3,4^. Recent researches related to anti-malarial drug development have focused plasmepsin inhibition largely^5–8^. Thus, the molecules showing inhibitory activities against plasmepsin enzymes can come out to be promising drug leads for treatment of malaria.

Cinchona alkaloids are cheap natural source of anti-malarial activity which provides opportunity towards development of new anti-malarial drug leads by synthetic modifications in their chemical structures. The anti-malarial activities of the four major alkaloids of this category follows the order Quinidine > Quinine > Cinchonidine > Cinchonine^9^. Thus, according to their anti-malarial activities, quinidine and quinine are the most interesting alkaloids to start with. But high anti-arrhythmic activities of these two alkaloids limit their use as regular anti-malarial drug because of cardiac risks even with smaller doses. Also, the drug resistance to quinine curbs the prospects of using this moiety in new drug leads. Therefore, cinchonidine remains the next potential molecule which can be modified structurally to enhance its anti-malarial activity.

The binding of drugs with proteins in blood is the key feature which determines the activity of the drugs^10^. Carbohydrate moiety, due to its good protein-binding traits serves as a protein-binding tool. Thus, addition of a carbohydrate moiety in a molecule improves binding of the molecule with proteins which further enhances its activity^11,12^. Triazole moiety possesses a unique property to interact with biological targets through dipole interactions and hydrogen-bonding, thus they serve as suitable pharmacophore to enhance biological activity of a molecule^13^.

**Figure 1:**
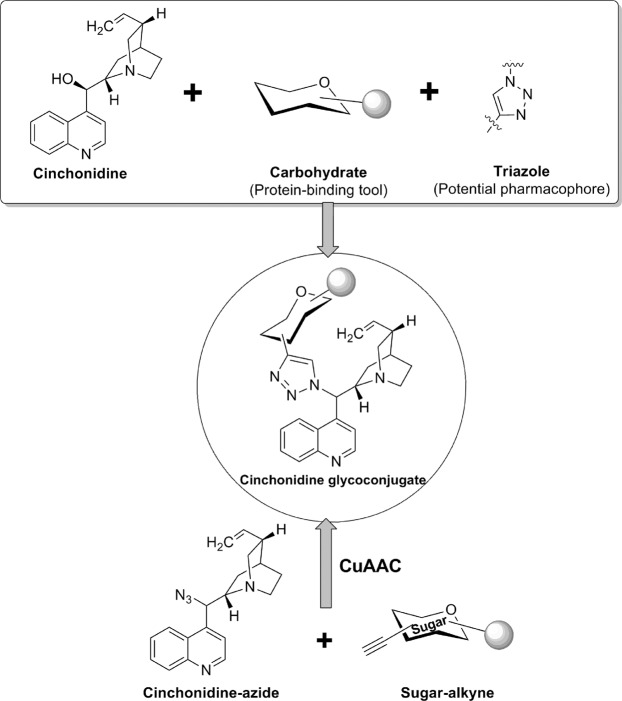
Rationale of development of triazolyl glycoconjugates of cinchonidine.

With a motive to develop novel cinchona alkaloid conjugates with anti-malarial activity, we preferred cinchonidine as the suitable alkaloid. Carbohydrate scaffold was chosen as a potential protein-binding moiety and 1,2,3-triazole was taken as suitable pharmacophore spacer to enhance activity of the resulting molecule^11,12^. The designed molecules containing these three moieties were selected as cinchonidine glycoconjugates with a triazole linker. Cu-Catalyzed click reaction is a facile and high yielding approach for 1,3-dipolar cycloaddition of organic azides and terminal alkynes which goes well with carbohydrate moiety and has produced very interesting glycosyl triazoles with various applications^14^. Thus, in continuation of our previous experience on click chemistry in glycoscience,^15–27^ this reaction was chosen for cycloaddition reaction of cinchonidine-derived azide and sugar-derived terminal alkynes to achieve our designed target molecules (Fig. 1).

## Results and Discussion

**Figure 2:**
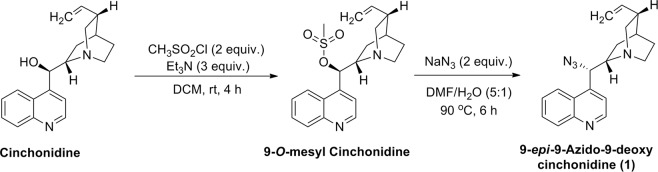
Synthesis of Cinchonidine azide derivative **1**.

The strategy for synthesis of cinchonidine-glycoconjugates with a triazole linker was initiated with the synthesis of azido-derivative of cinchonidine **1**. The free hydroxy group at C-9 of cinchonidine was chosen to be converted into azide group. Simple mesylation of the hydroxy group by treatment with methanesulphonyl chloride in presence of triethylamine afforded *O*-mesylated cinchonidine derivative which was as such subjected to heating with sodium azide in aqueous DMF and afforded 9-*epi*-9-azido-9-deoxycinchonidine **1**^28^. The azido derivative **1** was characterized by NMR, MS, and IR spectroscopy (Fig. 2).

**Figure 3:**
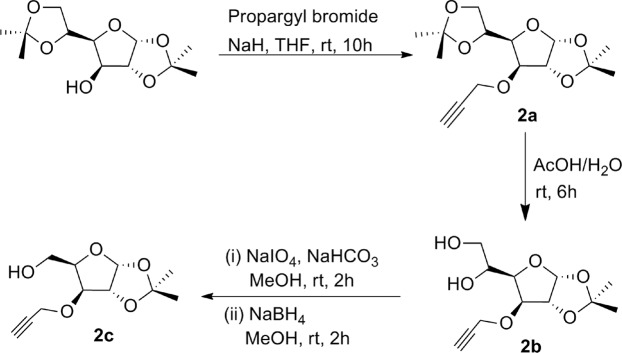
Synthesis of glycosyl *O*-propargyl ethers **2a–c**.

*O*-Propargyl ether derivatives of orthogonally protected carbohydrates **2a–j** were chosen as the appropriate glycosyl alkynes because of their easy synthesis and almost intact sugar architecture in their structure. The synthesis of sugar *O-*propargyl ethers **2a–c** was commenced with the orthogonal acetonide protection of D-glucose according to the standard procedures described in literature^29^. 3-*O*-Propargylation of acetonide protected glucose using propargyl bromide in THF in presence of NaH at room temperature for 10 hours afforded respective 3-*O*-propargyl ether derivative **2a** according to the procedure described in literature (Fig. 3)^30^. Treatment of compound **2a** with 70% aqueous acetic acid resulted into selective deprotection of isopropylidine group to furnish diol derivative **2b**^30^. Another modification was done in diol **2b** by reaction with sodium *meta*periodate in presence of NaHCO_3_ followed by reduction with sodium borohydride to afford derivative **2c** (Fig. 3)^30^.

**Figure 4:**
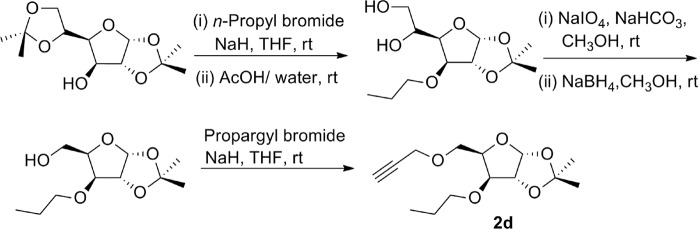
Synthesis of glycosyl *O*-propargyl ether **2d**.

Synthesis of glycosyl alkyne **2d** was again started from acetonide protected glucose, which on reaction with *n*-propyl bromide in presence of NaH followed by treatment with 50% aqueous acetic acid afforded respective diol derivative^31^. Resulted diol was subjected to oxidation with sodium *meta*periodate in presence of NaHCO_3_ followed by reduction in presence of sodium borohydride and at last the hydroxy derivative thus obtained on propargylation with propargyl bromide in presence of sodium hydride gave high yield of respective glucose derived *O*-propargyl derivative **2d** (Fig. 4).

**Figure 5:**
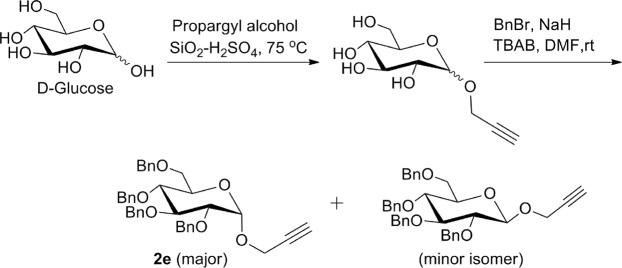
Synthesis of glycosyl *O*-propargyl ether **2e**.

Synthesis of 2,3,4,6-tetra-*O*-benzyl-1-*O-*propargyl-α-D-glucopyranose **2e** was achieved by a two-step reaction starting from D-glucose. Selective propargylation of anomeric hydroxy group of D-glucose by heating with propargyl alcohol in presence of SiO_2_-H_2_SO_4_ at 75 °C afforded 1-*O*-propargyl-α-D-glucopyranose which on further treatment with benzyl bromide in presence of sodium hydride and TBAB, was converted into an α/β- mixture of 2,3,4,6-tetra-*O*-benzyl-1-*O*-propargyl-D-glucopyranose. The α-isomer **2e** was separated as major isomer through column chromatography (Fig. 5)^30^.

**Figure 6:**
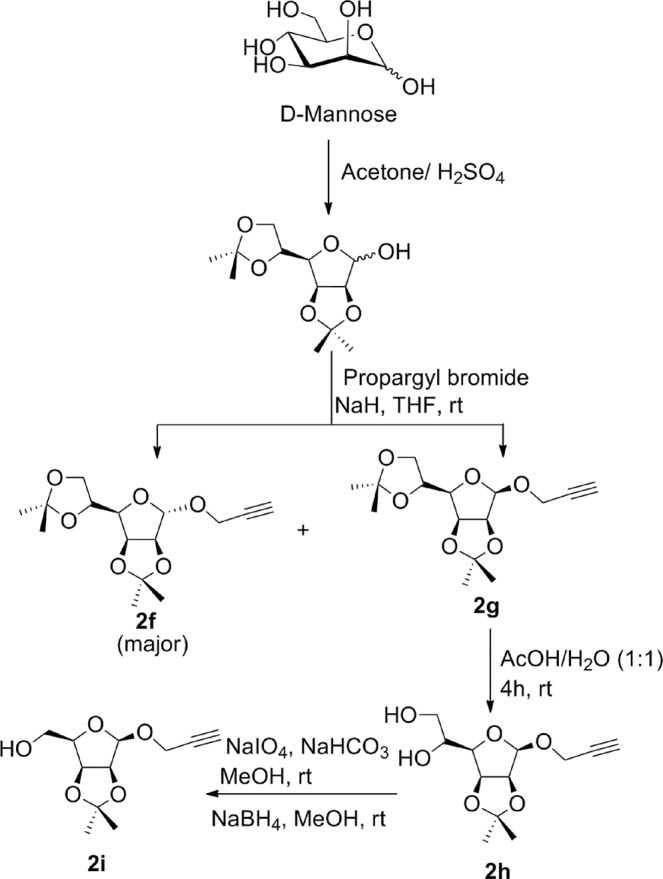
Synthesis of mannosyl *O*-propargyl ethers **2f–i**.

To synthesize *O*-propargyl ether derivatives of D-mannose **2f–i**, first of all, D-mannose was subjected to orthogonal acetonide protection in presence of sulphuric acid and acetone^29^. 2,3;5,6-Di-*O*-acetonide protected mannose was treated with propargyl bromide in presence of sodium hydride to afford *O*-propargyl derivative as a mixture of α- and β- isomers. These isomers were separated by column chromatography to afford pure 2,3;5,6-di-*O*-isopropylidine-1-*O*-propargyl-*α*-D-mannofuranose **2f** and 2,3;5,6-di-*O*-isopropylidine-1-*O*-propargyl-*β*-D-mannofuranose **2g**. These isomers were differentiated on the basis of their ^1^H and ^13^C NMR. In ^1^H NMR spectra of *α*-derivative **2f** the anomeric proton appeared at *δ* 5.16 ppm as singlet whereas, the anomeric proton of *β*-isomer **2g** appeared at *δ* 4.99 ppm as doublet which was confirmed through literature^30^. Also, in ^13^C NMR spectra of α-isomer **2f**, the anomeric carbon peak appeared at 104.8 ppm whereas, for *β*-isomer **2g**, it appeared at 100.4 ppm. *O*-Propargyl ether derivative **2f** was subjected to selective deprotection by treatment with 50% acetic acid-water to afford diol derivative 2,3-*O*-isopropylidine-1-*O*-propargyl-β-D-mannofuranose **2h**^30^. The selective oxidation of compound **2h** with sodium metaperiodate in presence of sodium bicarbonate afforded respective aldehyde derivative which at the end on reduction with sodium borohydride afforded respective propargylated mannose derivative **2i**^30^ (Fig. 6).

**Figure 7:**
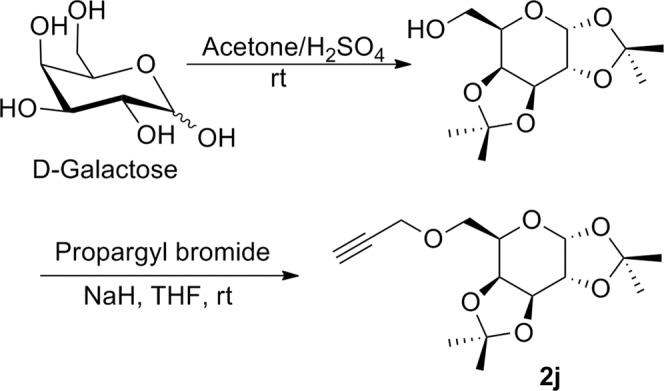
Synthesis of galactosyl *O*-propargyl ether **2j**.

The *O*-propargyl derivative of *α*-D-galactose was synthesized by reaction of orthogonally protected *α*-D-galactose with propargyl bromide in presence of sodium hydride resulting into formation of 2,3;4,5-di-*O*-isopropylidine-1-*O*-propargyl-*α*-D-galactopyranose **2j** (Fig. 7).

**Figure 8:**
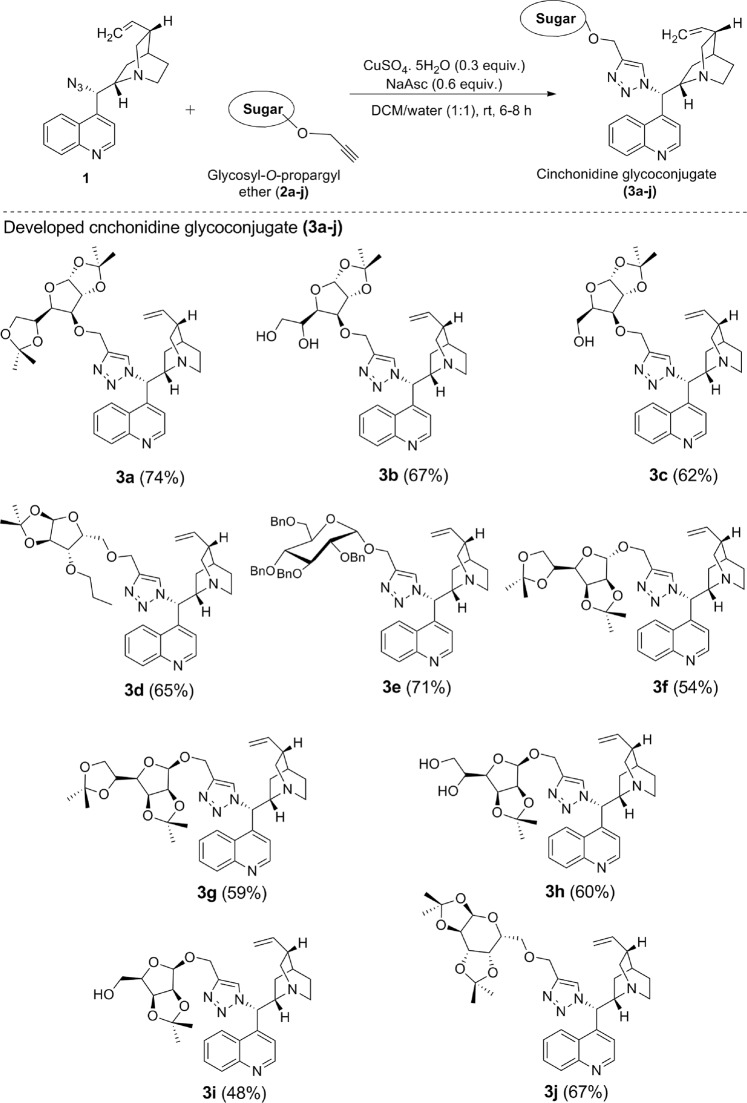
Synthesis of cinchonidine glycocnjugates (**3a–j)** from the respective glycosyl-*O*-propargyl ether **(2a–j)** and yields referred in % reported after purification by column chromatography (SiO_2_).

The synthesis of our target molecules **3a–j**, *i.e*. triazolyl glycoconjugates of cinchonidine, was achieved by Cu-catalyzed azide-alkyne cycloaddition of 9-epi-9-azido-9-deoxycinchonidine **2** and ten different glycosyl-*O*-propargyl ether derivatives. CuSO_4_ and sodium ascorbate mixture was taken as the catalyst system to generate Cu(I)-catalyst *in situ*. Both the starting compounds were found to be soluble in dichloromethane, thus DCM-water (1:1) was taken as suitable solvent for the reaction. A 6–8 hours reaction at room temperature gave good yields of final products **3a–j** (Fig. 8**)** which were characterized by ^1^H & ^13^CNMR, MS.

### Molecular docking of developed cinchona glycoconjugates

For the docking experiment Plasmepsin II (PDB ID 1LEE, PLM II), the first protein from protozoan parasite of the genus Plasmodium reported to be crystallized, considered as it is directly associated with the hemoglobin degradation which occurs during the malarial fever and parasite growth. It is an aspartic protease enzyme which initiates the degradation procedure and further is carried out via the affect of series of proteases in an acidic digestive vacuole. Plasmepsin I and II are the initiator of this sequential process which are homologous enzymes of 73% similar identical amino acid sequence. In the process an initial attack on the hemoglobin chain between residues 33 and 34 occurs followed by cleavage by the cysteine protease falcipain leading to degradation of hemoglobin.

Both of the plasmepsins are capable of making the initial cleavage in between Phe33 and Leu34 of the hemoglobin chain^32,33^ which leads to unraveling of hemoglobin molecule allowing proteolysis. In nineteen-nineties, a bunch of reports have the conclusion that inhibition of PLM II are lethal against cultured malarial parasites^34–40^. The used target complex is of PLM II from P. falciparum with new inhibitor having Phe-Leu core, which is reported to provide a view of the possible conformation of the enzyme when the natural substrate hemoglobin is bound and this strategy was focused in the experiment for achieving more reliable and justified docking results.

Structurally, PLM II has characteristic folding of eukaryotic aspartic proteases found in mammalian enzymes to the fungal enzymes and is a single chain of 329 amino acids along with two folded topologically similar *N*- and *C*-terminal domains constructed a binding cleft. Along the bottom of the binding cleft two domains contact each other and a catalytic dyad is formed of Asp34 and Asp214. Further a single hairpin structure, known as the ‘flap’, lies perpendicular over the binding cleft also reported to take part in the interaction with substrates. Moreover, a characteristic six-stranded interdomain β-sheet constructed by the amino and carboxyl ends of the polypeptide chain helped to anchor the domains together. The binding cavity is lined with water molecules alternatively positioned depending on the amino group present. As the synthesized molecular set, a triazolyl complex of cinchonidine and glycol-conjugate derivatives are a totally new class of chemical, the target found to good fit for docking study as the conformation of the enzyme typically depicted the orientation when the natural substrate hemoglobin is bound to it.

Docking was preferred for the virtual interaction estimation of the synthesized cinchonidine glycoconjugates with plasmepsin. The malarial target protein plasmepsin from *P. falciparum* in complex with inhibitor RS367 (PDB ID: 1LEE) was obtained from the RCSB protein data bank (PDB) having a resolution of 1.9 A^41^.

Auto Dock 1.5.6 software (ADT)^42,43^ and AutoDock Vina^44^ were used to investigate the interactability in terms of binding affinity (Kcal/mol) and the outcomes were compared in binding affinity score for best-docked conformation. The structures of the molecules were drawn by Chemdraw ultra 8.0^45^ and converted to the 3D structure using Chemdraw 3D. Finally the set of ligands and fragments were prepared by optimizing through molecular mechanics, then semi empirical method PM3 finally through DFT theory using Gauss 09 and converted to readable format for the ADT interface as pdb.

Molecular fragmentation was done according to the rules of DAIM stated. A fragment is defined to be the set of atoms connected by no easily breakable bonds avoiding generation of very small moieties and completion of the fragment with H or CH_3_ depending on the bonds present in the whole molecule^46^. The common structure is the cinchonidine ring structure in the fragments and the rest set of fragments is the glycosyl structure synthetically added to it. Fragment ligands were also prepared by DFT b3lyp/6-311 g method of optimization using Gauss 09 for achieving the best possible 3D orientation. The fragment docking has been done using AutoDock and Vina both. The optimized structures and IR spectra of fragments generated by Gauss have been depicted in Supporting Information (Table S1).

Docking was performed using combined energy evaluation through pre-calculated grids of affinity potential employing various search algorithms to find the suitable binding position for a ligand on a given protein (LOX) for both AutoDock and Vina docking softwares. All rotatable bonds in the ligands were kept free to allow flexible docking. Grid size was set to 50 × 50 × 50 grid points (x, y and z), with spacing between grid points kept at 0.375 Å. The grid box was generated using the axis details from ligand explorer for specification and precision controlling center grid box 30.969, 26.769, and 17.822 (x y and z)^41^. The Lamarckian genetic algorithm was chosen to search for the best conformers. Standard docking protocol was applied.

For AutoDock a set of two hundred fifty independent docking runs for each fragment were generated using genetic algorithm search. The outcomes of results were analyzed by AutoDock analyzer and the complex.pdb file was investigated in the discovery studio visualize for better interpretability and the result regards the classical hydrogen bond interaction between the macromolecule and the ligand moiety. Docking method used is the standard method reported and the best interacting conformer with least binding energy is the final result.

For AutoDock Vina, a set of 10 independent docking runs for 15 times for each fragment and a set of conformers were generated to produce a population of 150 using genetic algorithm search. The outcomes of results were analyzed by AutoDock analyzer using the PDBQT files and the result regards the classical hydrogen bond interaction between the macromolecule and the ligand moiety. Docking method used is the standard method reported and the best interacting conformer with least binding energy has been concluded as the final result.

The whole set of ligands was prepared by DFT b3lyp/6-311 g method for achieving the most stable 3D orientation in space and further subjected for docking and it is validated by the use of IR spectra of each ligand. All the optimized structures of cinchonidine glycoconjugates and respective IR spectra generated by Gauss have been reported in Supporting Information (Table S2).

The optimization of ligand **3e** had only been possible using semi empirical calculation, not using DFT β3LYP/6-311g(d,p) method due to the bulky group substituents. Huge molecular system caused unsupportive to the proper calculation. So the moiety was discarded for the comparable docking study in the group as the structures of the other ligands were optimized through the DFT calculations.

### Fragment docking results using AutoDock

The whole molecule docking was carried out using AutoDock but the results were unsatisfying, under acceptability and erroneous; so, discarding that molecules were subjected for fragmentation study.

**Figure 9:**
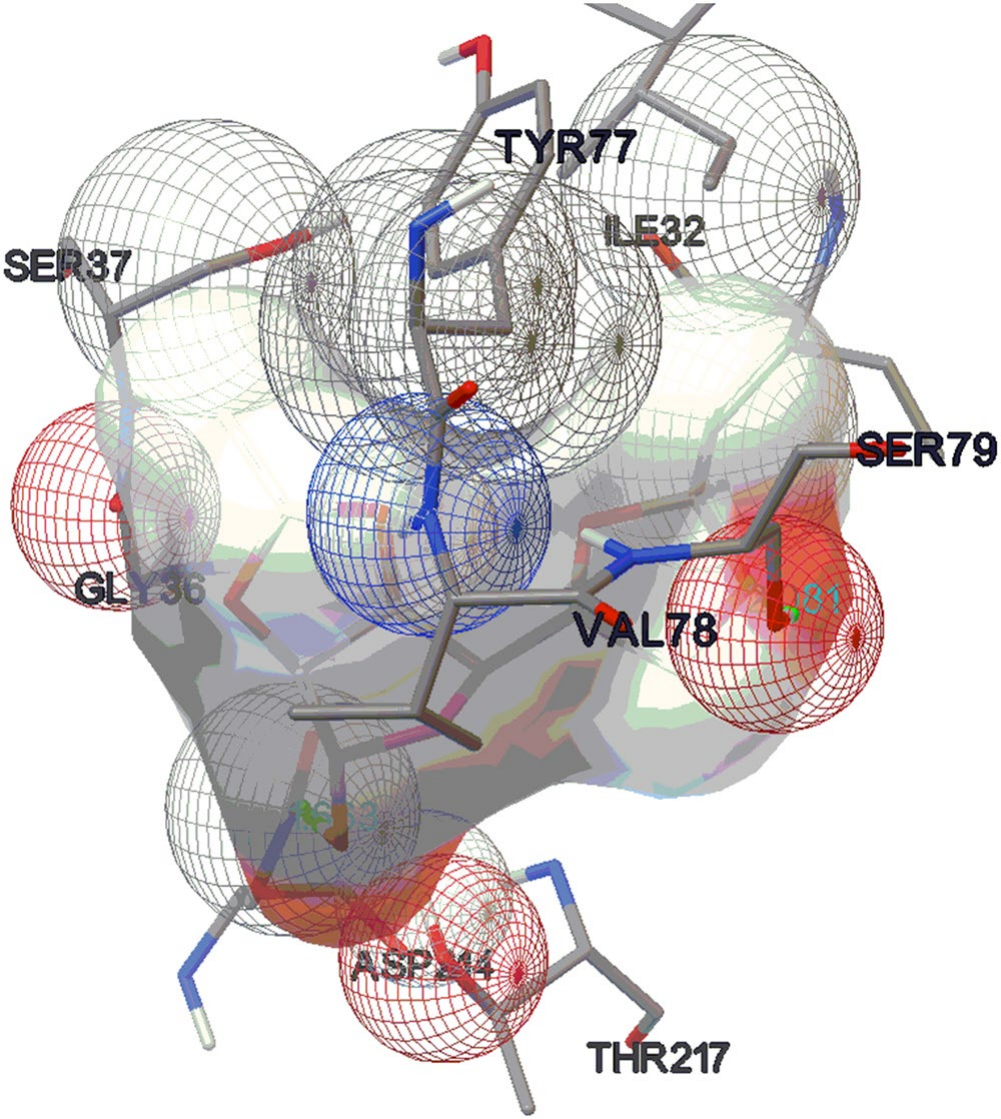
Molecular docking picture of fragment **3f** (AutoDock).

Fragment docking was done using AutoDock following the standard protocol. The results have been summarized in Table 1. The results revealed that the key conserved hydrogen bonds are between the fragmented ligands and the binding-cavity residues, notably with the flap residues VAL78 and SER79, the catalytic dyad ASP34 and ASP214 and the residues SER218 and GLY36. The set of amino acids showing the H-bond interaction with the ligands are the most interacting residues of the enzyme. The co-crystal R367 is reported to show interaction with this amino acid sets which is an anti-malarial under trial drug. Fragmented ligand **3f (**Table 1, entry 6] has the highest binding value with −5.8 Kcal/mol and 2 H-bond interactions to the ASP214 and SER79 amino acid residues (Fig. 9). Next, ligands **3a**, **3b, 3g**, **3h**, and **3j** (Table 1, entry 2, 3, 7, 8 and 10) have comparable binding energy values in between the range of −5.46to −5.20 Kcal/mol with one or two H bond interactions. The results show that sugar fragments have a good potential in the interaction which in turn will be helpful in enhancing the binding with the targeted activity. The pictures of fragment molecular docking using AutoDock has been depicted in Supporting Information (Figs. S1–S10).

**Table 1:** Results of fragment docking using AutoDock.

Entry	Molecular fragment	Binding energy (K cal/ mol)	Number of H-bonds	H-bond length (Ǻ)	Amino acid
1	Cm	−6.1	2	1.858	VAL78
				1.84	THR217
**2**	**3a**	**−5.35**	**1**	**1.938**	**ASP34**
**3**	**3b**	**−5.46**	**2**	**2.018**	**ASP214**
				**2.012**	**SER79**
4	**3c**	−4.9	2	1.799	ASP303
				1.811	SER79
5	**3d**	−4.9	2	1.886	VAL78
				2.146	SER79
6	**3f**	**−5.8**	**2**	**1.863**	**ASP214**
				**2.081**	**SER79**
7	**3g**	−5.2	2	1.872	GLY216
				2.144	SER218
8	**3h**	−5.23	2	1.915	SER79
				2.013	ASP214
9	**3i**	−4.71	2	2.22	VAL78
				1.969	ASP34
**10**	**3j**	**−5.39**	**1**	**2.137**	**VAL78**

### Fragment docking results using AutoDock Vina

**Figure 10:**
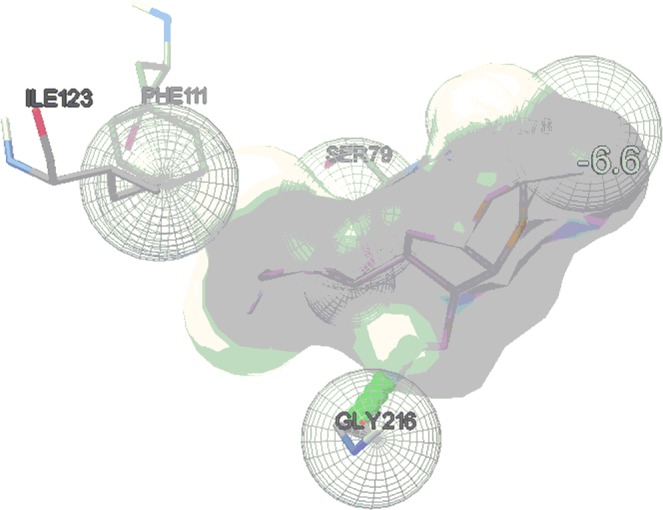
Molecular docking picture of fragment **3a** (Vina).

For comparative study, the fragmented ligands, which were prepared through DFT b3lyp/6-311g method using Gauss 09, were subjected to the Vina docking. 150 Set of docked conformers were evaluated for the least binding energy along with the highest number of possible H-bond interactions which was specific, reliable and reproducible for a number of times. Those conformers are reported for each fragment ligand. The results have been summarized in Table 2. These fragmented ligands conserved hydrogen bonds notably with the flap residues Val78 and Ser79 of the enzyme and with the residues GLY216, GLY36, THR217 that are in proximity to the catalytic dyad. Fragmented ligand **3a** (Table 2, entry 2) showed the best binding energy of value −6.6 Kcal/mol with 1 H-bond interaction with GLY216 (Fig. 10), followed by ligand **3f** (Table 2, entry 6) having the binding energy value of −6.3 with 2 H-bond interactions with SER79 and THR217 respectively. Next, ligand **3b**, **3c**, **3g**, **3h** and **3j** (Table 2, entry 3, 4, 7, 8 and 10) showed a comparable binding energy values in between the range of −5.9 to −5.5 Kcal/mol with one or two H-bond interactions. The pictures of fragment molecular docking using AutoDock Vina have been depicted in Supporting Information (Figs. S11–S20).

**Table 2:** Results of fragment docking using AutoDock Vina.

Entry	Molecular fragment	Binding energy (K cal/ mol)	Number of H-bonds	Amino acid
1	Cm	−5.4	1	VAL78
**2**	**3a**	**−6.6**	**1**	**GLY216**
3	**3b**	−5.9	1	GLY216
4	**3c**	−5.5	1	SER79
5	**3d**	−5.1	2	GLY216, VAL78
**6**	**3f**	**−6.3**	**2**	**SER79, THR217**
7	**3g**	−5.8	1	SER79
8	**3h**	−5.5	1	VAL78
9	**3i**	−4.8	1	SER79
10	**3j**	−5.9	1	GLY216

### Whole molecular docking results using AutoDock Vina

**Figure 11:**
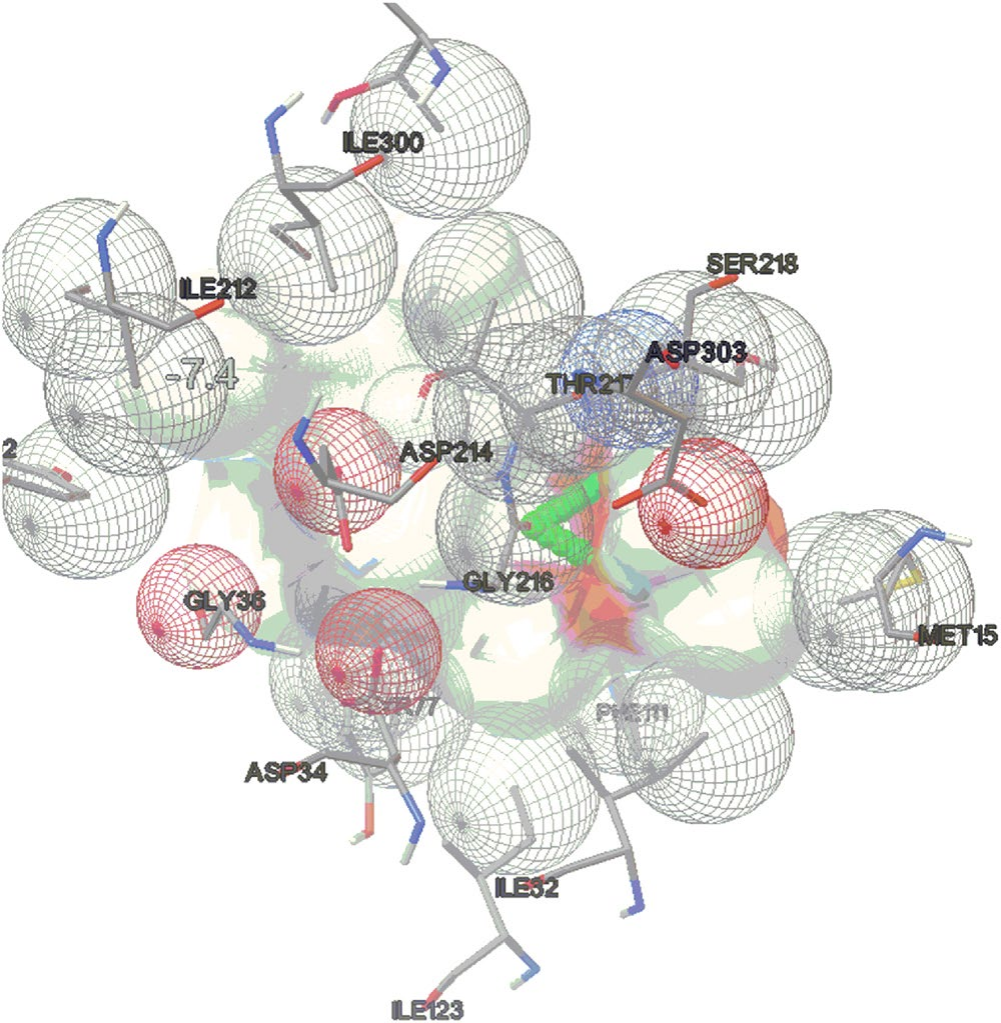
Molecular docking picture of whole molecule **3g** (Vina).

Whole moiety docking was done using Vina for the evaluation of binding energy and interaction pattern for the total set of the molecules. The binding energy values and interaction pattern are comparable. The results have been summarized in Table 3. These complexes conserved hydrogen bonds notably with the flap residues VAL78 and SER79 of the enzyme and with the residues GLY216, SER215, GLY36, THR217 besides, catalytic dyad residues ASP34 and ASP214. Ligand **3g** (Table 3, entry 6) has shown highest bonding energy of −7.4 Kcal/mol and 2 H-bond interactions with GLY216 (Fig. 11). Next, ligand **3c** (Table 3, entry 3) and **3h** (Table 3, entry 7) followed the order of the highest binding energy with values of −7.1 and −6.5 Kcal/mol respectively along with 1 H-bond interaction each. Ligand **3d** (Table 3, entry 4) has exceptionally shown 5 H-bond interactions with VAL78, GLY216 and THR217 which are the important amino acids of the catalytic active site having binding energy value of −4.9 Kcal/mol. The H-bond has been formed mainly between the triazole or glycosylated part with the catalytic site constituting amino acids which clearly states that the attached extent with the cinchonidine has been fruitful for increasing the activity. The pictures of whole molecular docking using AutoDock Vina have been depicted in Supporting Information (Figs. S21–S29).

**Table 3:** Results of whole molecular docking using AutoDock Vina.

Entry	Molecule	Binding energy (K cal/ mol)	Number of H-bonds	Amino acid
1	**3a**	−3.6	2	GLY216, SER215
2	**3b**	−5.2	2	GLY216, GLY38
**3**	**3c**	**−7.1**	**1**	**VAL78**
**4**	**3d**	**−4.9**	**5**	**THR217, GLY216, VAL78**
5	**3f**	−5.3	2	GLY217, SER79
**6**	**3g**	**−7.4**	**2**	**GLY216**
**7**	**3h**	**−6.5**	**1**	**SER79**
8	**3i**	−5.5	2	SER79, ASP34
9	**3j**	−3.9	3	GLY216, SER79, GLY36

### Validation of the docking method

**Figure 12:**
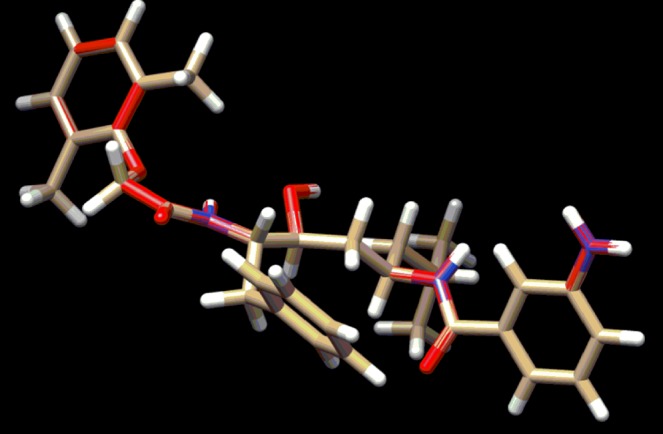
Superimposed structure of the co-crystal reported in RCSB PDB with the protein before and after docking study.

The validation of docking method has been done using superimpose fitting method by Chimera 1.11.2 software. The superimpose fitting has been done using the whole atom set of the co-crystal system using the raw pdb file reported over the docked pose of it after the docking study applying the three methods. The result has been the fitting of value within 2Ǻ of acceptability range (Fig. 12) with the value of 0.515 Ǻ variation. This proves that the methods applied are correct. On repetition reproducibility and specificity of the result have been achieved over a population set of 150. So the method is well validated and acceptable.

**Figure 13:**
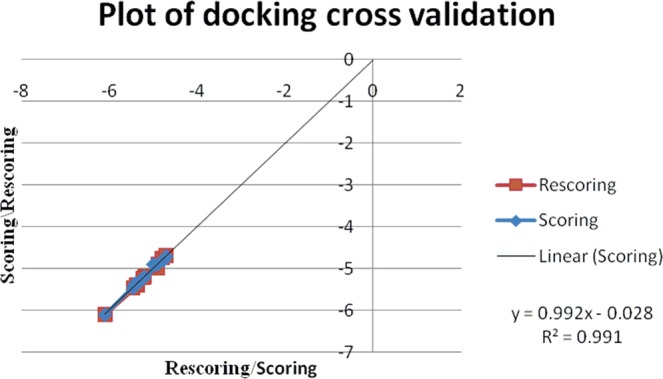
Plot of docking score/rescoring vs rescoring/scoring for fragment docking using AutoDock.

**Figure 14:**
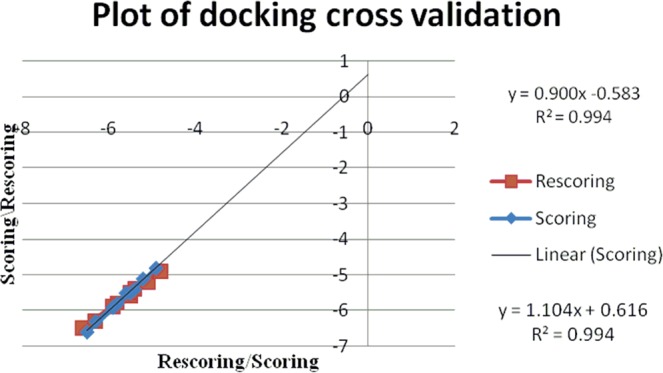
Plot of docking score/rescoring vs rescoring/scoring for fragment docking using AutoDock Vina.

**Figure 15:**
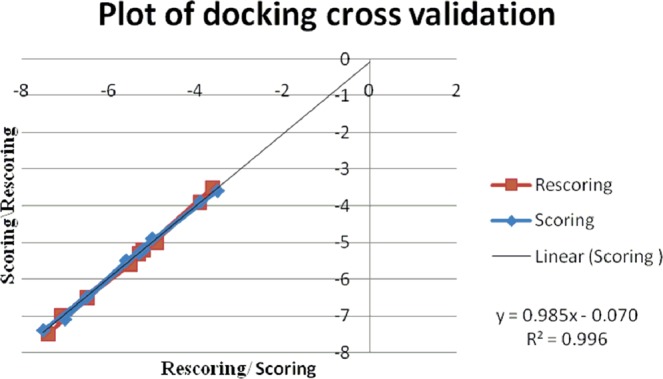
Plot of docking score/rescoring vs rescoring/scoring for whole molecular docking using AutoDock Vina.

The docking procedure has been cross validated after the docking method validation through super-imposition method of co-crystal structures, by repeating the procedure for another cycle (Tables 4–6). The second cycle score has been plotted as rescoring on y-axis and the score has been taken in the x-axis. The regression value (R^2^) for each docking experiment is near to value 1 which within the acceptable limits with good results along with the slope value difference of the straight lines are less than 0.1 which ensures the reliability of the method. The values clearly indicate the reliability, repeatability and reproducibility of the experiment carried out (Figs. 13–15).

**Table 4:** Re-docking validation for fragment docking using AutoDock.

Molecular fragment	Binding energy (K cal/ mol)		Number of H-bonds	Amino acid
	Docking	Re-docking		
Cm	−6.1	−6.1	2	VAL78
				THR217
**3a**	−5.35	−5.4	1	ASP34
**3b**	−5.46	−5.46	2	ASP214
				SER79
**3c**	−4.9	−5	2	PHE 16
				ASP303
**3d**	−4.9	−4.9	2	VAL78
				SER79
**3f**	−5.8	−5.76	2	ASP214
				SER79
**3g**	−5.2	−5.2	2	GLY216
				SER218
**3h**	−5.23	−5.23	2	SER79
				ASP214
**3i**	−4.71	−4.7	2	VAL78
				ASP34
**3j**	−5.39	−5.39	1	VAL78

**Table 5:** Re-docking validation of fragment docking using AutoDock Vina.

Molecular fragment	Binding energy (K cal/ mol)		Number of H-bonds	Amino acid
	Docking	Re-docking		
Cm	−5.4	−5.4	1	VAL78
**3a**	−6.6	−6.5	1	GLY216
**3b**	−5.9	−5.9	1	GLY216
**3c**	−5.5	−5.6	1	SER79
**3d**	−5.1	−5.2	2	GLY216, VAL78
**3f**	−6.3	−6.3	2	SER79, THR217
**3g**	−5.8	−5.8	1	SER79
**3h**	−5.5	−5.5	1	VAL78
**3i**	−4.8	−4.9	1	SER79
**3j**	−5.9	−5.9	1	GLY216

**Table 6:** Re-docking validation of whole molecule docking using AutoDock Vina.

Molecule	Binding energy (K cal/ mol)		Number of H-bonds	Amino acid
**3a**	−3.6	−3.5	2	GLY216, SER215
**3b**	−5.2	−5.2	2	GLY216, GLY38
**3c**	−7.1	−7	1	VAL78
**3d**	−4.9	−5	5	THR217, GLY216, VAL78
**3f**	−5.3	−5.3	2	GLY216, SER79
**3g**	−7.4	−7.5	2	GLY216
**3h**	−6.5	−6.5	1	SER79
**3i**	−5.5	−5.6	2	SER79, ASP34
**3j**	−3.9	−3.9	3	GLY216, SER79, GLY36

## Conclusions

Ten novel molecular sets consisting of cinchonidine, triazole ring and carbohydrate scaffolds were designed and synthesized using Cu-catalyzed azide-alkyne cycloaddition reaction of 9-*epi-*9-azido-9-deoxycinchonidine with ten different glycosyl *O-*propargyl ethers. Developed cinchonidine glycoconjugates were subjected to docking studies for the evaluation of the interaction probability for anti-malarial activity with appreciable values of inhibitory effect. The molecular set has been subjected to both fragment docking and whole molecular docking using AutoDock and AutoDock Vina softwares. The detailed comparative docking study revealed that moieties are showing H-bond interaction at the target site where the anti-malarial active co-crystal moiety, R367 has been reported to show its interaction with amino acid residues of plasmepsin enzyme in the crystal structure. Therefore, it has a strong probability that the molecular set can show anti-malarial property. According to the results of fragment docking using AutoDock, fragmented ligand **3f** has the highest binding value with −5.8 Kcal/mol followed by ligands **3a**, **3b**, **3g**, **3h** and **3j** which have comparable binding energy values within the range of −5.39 to −5.2 Kcal/mol. In fragment docking using Vina, fragmented ligand **3a** has shown the best binding energy of value −6.6 Kcal /mol, followed by ligand **3f** with value of −6.3 with 2 H-bond interactions with SER79 and THR217. Fragmented ligands **3b**, **3c**, **3g**, **3h** and **3j** have a comparable binding energy values in between the range of −5.9 to −5.5 Kcal/mol. In the whole molecular docking using Vina, ligand **3g** has shown highest bonding energy of −7.4 Kcal/mol. Next, ligand **3c** and **3h** followed the order of the highest binding energy value between range of −7.1 and −6.5 Kcal/mol respectively. From the comparative docking studies it can be concluded that glycoconjugate fragmented ligands **3a** and **3b** are the best moieties with least binding energy values and greater number of H-bond interaction with the active site of the macromolecule. Besides, as a whole molecular system, ligand **3g** is also a considerable moiety for anti malarial activity. Apart from these, fragmented as well as whole molecular ligands **3c** and **3h** have shown good binding energy with promising macromolecular interaction.

## Materials and Methods

### General

All the reactions were performed under argon atmosphere using anhydrous solvents. All the reagents and solvents used were of analytical grade. Glasswares were dried in an oven at 100 °C for one hour and cooled in a desiccator before use. 60 F254 Silica gel pre-coated aluminum plates were used for thin layer chromatography (TLC) and spots were located either under a UV lamp (*λmax* = 254 nm) or by charring after 5% H_2_SO_4_-MeOH solution spray. ^1^H and ^13^C NMR were recorded at 500 MHz and 125 MHz, respectively. Chemical shifts given in ppm are relative to that of TMS as internal standard; *J* values are given in Hz. IR spectra were recorded as Nujol mulls in KBr pellets.

### Synthesis of 9-*epi-*9-azido-9-deoxycinchonidine (1)

Cinchonidine (3.0 g, 10.19 mmol) was taken in anhydrous DCM (40 ml) in a round bottom flask and the suspension was cooled to 0 °C in an ice bath under argon atmosphere. Triethyl amine (3.09 g, 4.3 ml, 30.57 mmol) and methane sulphonylchloride (2.33 g, 1.6 ml, 20.38 mmol) were added to the cooled solution. After addition, the temperature was raised to room temperature and the mixture was continued to stir at room temperature for 4 hours. Completion of reaction was monitored by TLC. The mixture was then washed with water 2–3 times, dried over anhyd. Na_2_SO_4_ and concentrated under reduced pressure to afford crude mesylated compound (3.8 g). This crude compound was dissolved in 6 ml of DMF/ water (5:1) and sodium azide (1.32 g, 20.38 mmol) was added to it. The mixture was heated at 90 °C for 8 hours. The reaction mixture was then cooled to room temperature, diluted with water and extracted with ethyl acetate. The organic layer was dried over anhyd. Na_2_SO_4_ and concentrated under reduced pressure. Purification was done by column chromatography (SiO_2_) using gradient mixture of DCM/methanol (49:1) to afford 2.5 g of pure compound **1**. Light brown sticky mass, yield 76.8%; R_*f*_ = 0.5 (5% MeOH/DCM); IR (KBr): *ν*_max_ 2926, 2865, 2100, 1636, 1591, 1509, 1454, 1260, 1114, 820, 762, 619 cm^−1^; ^1^H NMR (500 MHz, CDCl_3_): δ 8.89 (d, *J* = 4.5 Hz, 1H), 8.19–8.12 (m, 2H), 7.71–7.68 (m, 1H), 7.59–7.56 (m, 1H), 7.35 (d, *J* = 3.5Hz, 1H), 5.70–5.67 (m, 1H), 5.08 (d, *J* = 9.5Hz, 1H), 4.95–4.89 (m, 2H), 3.29–3.15 (m, 3H), 2.85–2.77 (m, 2H), 2.22 (bs, 1H), 1.47–1.19 (m, 4H), 0.69–6.65 (m, 1H); ^13^C NMR (125 MHz, CDCl_3_): δ 149.8, 148.5, 142.0, 141.1, 130.4, 129.3, 127.0, 126.4, 122.8, 120.1, 114.3, 59.4, 55.7, 40.7, 39.1, 29.4, 27.6, 26.9, and 25.9 ppm.

### Synthesis of glycosyl alcohols and *O*-propargyl ethers

Glycosyl *O-*propargyl ethers **2a**, **2b, 2c**, **2e**, and **2j** were synthesized according to standard procedure described in literature^29^.

### 1,2-*O-*isopropylidene-3-*O-*propyl-a-D-xylofuranose

Diol **(**600 mg, 2.28 mmol) was dissolved in methanol (20 ml) and NaIO_4_ (734 mg, 3.43 mmol) and NaHCO_3_ (211 mg, 2.51 mmol) was added to it. The mixture was stirred a room temperature for 2.5 hours. The reaction mixture was then filtered through celite bed and the filtrate was concentrated under reduced pressure to obtain the respective aldehyde (480 mg). This crude compound was dissolved in methanol (20 ml) and NaBH_4_ (129 mg, 3.42 mmol) was added to it. The mixture was stirred at room temperature for 4 hours and the completion of reaction was monitored by TLC. The reaction mixture was then concentrated at reduced pressure. The crude was dissolved in ethyl acetate and washed with water and brine, dried over anhyd. Na_2_SO_4_ and concentrated under reduced pressure. The crude was purified by silica gel column chromatography (230–400 mesh) using gradient mixture of *n*-hexane/ ethyl acetate (9:1) to afford 1,2-*O*-isopropylidene-3-*O*-propyl-a-D-xylofuranose in pure form (460 mg). Colourless oil, yield 87%; R_*f*_ = 0.5 (30% ethyl acetate/*n*-hexane); ^1^H NMR (500 MHz, CDCl_3_): δ 5.98 (d, *J* = 4.5 Hz, 1H), 4.57 (d, *J* = 3.5 Hz, 1H), 4.29–4.28 (m, 1H), 3.98–3.91 (m, 3H), 3.62–3.58 (m, 1H), 3.43–3.38 (m, 1H), 2.47 (s, 1H), 1.63–1.56 (m, 2H), 1.49 (s, 3H), 1.33 (s, 3H), 0.92 (t, *J* = 7.5 Hz, 3H); ^13^C NMR (125 MHz, CDCl_3_): δ 111.7, 105.1, 84.2, 82.4, 79.9, 72.1, 61.1, 26.8, 26.3, 22.9 and 10.5 ppm.

### 1,2-*O-*isopropylidene-5-*O-*propargyl-3-*O-propyl-a-D-xylofuranose* (**2d**)

1,2-*O*-isopropylidene-3-*O*-propyl-a-D-xylofuranose (190 mg, 0.82 mmol) was dissolved in anhydrous THF and the resultant solution was cooled to 0 °C in an ice bath under argon atmosphere. To this solution, NaH (39 mg, 1.63 mmol) was added portion wise under argon atmosphere and the resulting suspension was stirred at room temperature. After one hour, propargyl bromide (126 mg, 0.08 ml, 1.06 mmol) was added to it and the mixture was stirred at room temperature for 6 hours. Completion of reaction was monitored by TLC. The reaction mass was then quenched by adding methanol. The solution was then concentrated under reduced pressure and diluted with ethyl acetate, washed with water and brine, dried over anhydrous Na_2_SO_4_ and concentrated under reduced pressure. The crude thus obtained was purified by silica gel column chromatography (230–400 mesh) using gradient mixture of ethyl *n*-hexane /acetate (9:1) to afford pure compound **2d** (186 mg). Colourless oil, yield 84%; R_*f*_ = 0.5 (20% ethyl acetate/*n*-hexane); ^1^H NMR (500 MHz, CDCl_3_): δ 5.84 (d, *J* = 4.0 Hz, 1H), 4.77 (d, *J* = 4.0 Hz, 1H), 4.31–4.28 (m, 1H), 4.19–4.08 (m, 2H), 3.77–3.74 (m, 2H), 3.67–3.64 (m, 1H), 3.51–3.47 (m, 1H), 3.34–3.30 (m, 1H), 2.37–2.36 (m, 1H), 1.60–1.49 (m, 2H), 1.41 (s, 3H), 1.24 (s, 3H), 0.85 (t, *J* = 3.0 Hz, 3H); ^13^C NMR (125 MHz, CDCl_3_): δ 111.7, 105.1, 82.4, 82.3, 79.6, 79.1, 74.6, 72.2, 67.4, 58.6, 26.8, 26.3, 22.9 and 10.6 ppm.

#### 2,3;5,6-Di-O-isopropylidine-1-O-propargyl-α-D-mannofuranose (**2f**)

To a solution of 2,3;5,6-di-*O*-isopropylidine-α-D-mannofuranose (15.0 g, 0.083 mol) in anhydrous THF (200 ml) NaH (3.98 g, 0.166 mol) was added portionwise at 0 °C under argon atmosphere. After addition, the temperature was raised to room temperature and the mixture was allowed to stir for 30 minute at room temperature. Then, proprgylbromide (19.7 g, 1.57 ml, 0.166 mol) was added to the reaction mixture at the same temperature. The reaction was allowed to stir at room temperature for 18 hours and monitored by TLC for the completion. After completion of reaction, it was quenched by adding methanol under ice cold condition. The solution was then concentrated under reduced pressure and diluted with ethyl acetate. The solution was washed with water and brine, dried over anhydrous Na_2_SO_4_ and concentrated under reduced pressure to obtain a crude mixture of α- and β- isomeric forms. Separation of isomers was done by silica gel column chromatography (230–400 mesh) using gradient mixture of *n*-hexane/ethylacetate (9:1) to afford pure compound **2f** (α-anomeric product, 7.9 g). Colourless oil, yield 32%; R_*f*_ = 0.4 (20% ethyl acetate/*n*-hexane); ^1^H NMR (500 MHz, CDCl_3_): δ 5.16 (s, 1H), 4.78–4.77 (m, 1H), 4.62 (d, *J* = 6.0 Hz, 1H), 4.41–4.38 (m, 1H), 4.17–4.16 (m, 2H), 4.11–4.02 (m, 2H), 3.94 (dd, *J* = 4.0, 5.0 Hz, 1H), 2.43 (t, *J* = 2.0 Hz, 1H), 1.46 (s, 3H), 1.44 (s, 3H), 1.36 (s, 3H), 1.31 (s, 3H); ^13^C NMR (125 MHz, CDCl_3_): δ 112.6, 109.2, 104.8, 84.9, 80.6, 79.4, 78.8, 74.6, 73.0, 66.8, 54.0, 26.8, 25.8, 25.1 and 24.4 ppm.

#### 2,3;5,6-Di-O-isopropylidine-1-O-propargyl-β-D-mannofuranose (**2g**)

Separated from anomeric mixture following above procedure resulted to the isolation of compound **2g** in pure form (1.86 g, 11%); R_*f*_ = 0.8 (20% ethyl acetate/*n*-hexane); ^1^H NMR (500 MHz, CDCl_3_): δ 4.99 (d, *J* = 4.0 Hz, 1H), 4.74–4.72 (m, 1H), 4.65 (dd, *J* = 4.0, 2.0 Hz, 1H), 4.45–4.41 (m, 2H), 4.36–4.33 (m, 1H), 4.08–4.07 (m, 2H), 3.64 (dd, *J* = 3.5, 4.0 Hz, 1H), 2.44–2.43 (m, 1H), 1.53 (s, 3H), 1.43 (s, 3H), 1.36 (s, 3H), 1.34 (s, 3H); ^13^C NMR (125 MHz, CDCl_3_): δ 113.9, 109.3, 100.4, 79.8, 79.1, 78.7, 75.2, 73.3, 66.8, 56.8, 27.0, 25.6, 25.3, and 25.0 ppm.

#### 2,3-O-isopropylidene-1-O-propargyl-β-D-lyxofuranose (**2h**)

Compound **2f** (1.0 g, 3.35 mmol) was taken in 50% AcOH/water and stirred for 8 hours at room temperature. The mixture was then concentrated under reduced pressure, diluted with ethyl acetate, washed with water 3–4 times, dried over anhyd. Na_2_SO_4_ and concentrated under reduced pressure to obtain pure compound **2h** (730 mg). Yellowish oil, yield 86%; R_*f*_ = 0.4 (70% ethyl acetate/*n*-hexane); ^1^H NMR (500 MHz, CDCl_3_): δ 5.14 (s, 1H), 4.81–4.79 (m, 1H), 4.57 (d, *J* = 7.0 Hz, 1H), 4.12(d, *J* = 2.0 Hz, 2H), 3.95–3.88 (m, 2H), 3.81–3.78 (m, 1H), 3.65–3.62 (m, 1H), 3.46 (bs, 1H), 3.02 (bs, 1H), 2.42–2.41 (m, 1H), 1.42 (s, 3H), 1.28 (s, 3H); ^13^C NMR (125 MHz, CDCl_3_): δ 112.8, 104.7, 84.7, 79.9, 79.6, 79.0, 74.8, 70.0, 64.3, 54.1, 26.0 and 24.7 ppm.

#### 2,3-O-isopropylidene-1-O-propargyl-β-D-lyxofuranose (**2i**)

Compound **2h** (700 mg, 2.71 mmol) was taken in methanol in a round bottom flask and NaIO_4_ (870 mg, 4.06 mmol) and NaHCO_3_ (250 mg, 2.98 mmol) were added to it. The reaction mixture was allowed to stir for 2.5 hours at room temperature. After completion of reaction (monitored by TLC), the reaction mass was filtered through celite bed. The filtrate was concentrated under reduced pressure to obtained *2,3-O-isopropylidene-1-O-(2-propyn-1-yl)-α-D-lyxose-pentadialdo-1,4-furanose* (490 mg). Colourless oil, yield 80%; R_*f*_ = 0.6 (30% MeOH/DCM); ^1^H NMR (500 MHz, CDCl_3_): δ 9.53 (s, 1H), 5.28 (d, *J* = 2.0 Hz, 1H), 5.00 (s, 1H), 4.58–4.51 (m, 1H), 4.31 (s, 1H), 4.13–4.09 (m, 2H), 2.41–2.37 (m, 1H), 1.32 (s, 3H), 1.18 (s, 3H); ^13^C NMR (125 MHz, CDCl_3_): δ 197.4, 113.5, 105.1, 84.5, 84.3, 80.7, 78.5, 75.2, 54.3, 25.8 and 24.5 ppm.

The resulted aldehyde (490 mg, 2.16 mmol) was dissolved in methanol (15 ml) and NaBH_4_ (164 mg, 4.33 mmol) was added to it. The mixture was stirred at room temperature for 3 hours. The reaction mass was concentrated at reduced pressure and dissolved in ethyl acetate. The solution was washed with water and brine, dried over Na_2_SO_4_ and concentrated under reduced pressure. Crude mass thus obtained was purified by silica gel column chromatography using gradient mixture of *n*-hexane/ethylacetate (9:1) to afford pure compound 2i (386 mg). Yellowish oil, yield 78%; R_*f*_ = 0.4 (20% ethyl acetate/*n*-hexane); ^1^H NMR (500 MHz, CDCl_3_): δ 5.17 (s, 1H), 4.74–4.70 (m, 1H), 4.58 (d, *J* = 6.5 Hz, 1H), 4.18–4.11 (m, 2H), 4.04–4.01 (m, 1H), 3.88–3.84 (m, 2H), 2.58 (bs, 1H), 2.41–2.40 (m, 1H), 1.40 (s, 3H), 1.24 (s, 3H); ^13^C NMR (125 MHz, CDCl_3_): δ 112.8, 104.4, 85.1, 80.1, 80.0, 79.9, 74.8, 60.8, 54.0, 25.9 and 24.6 ppm.

### General procedure for synthesis of cinchonidine glycoconjugates

Azido cinchonidine **1** (1.0 equiv.) and glycosyl alkyne **2** (1.0 equiv.) were taken in DCM/water (1:1) in a round bottom flask and to it, CuSO_4_.5H_2_O (0.3 equiv.) and sodium ascorbate (0.6 equiv.) were added. The mixture was stirred at room temperature for 6–8 hours. After completion of reaction (monitored by TLC), the reaction mixture was filtered through filter paper and the filtrate was diluted with DCM, washed with water and brine, dried over anhyd. Na_2_SO_4_ and concentrated under reduced pressure. The crude was purified by silica gel column chromatography using gradient mixture of DCM/ MeOH to obtain pure compound **3**.

#### 9-Deoxy-9-(4-(1,2;4,5-di-O-isopropylidine-3-O-methyl-α-D-glucofuranos-3-yl)-1H-1,2,3-triazole-1-yl]-epi-cinchonidine (3a)

Light brown solid, yield 74%; R_*f*_ = 0.5 (5% MeOH/DCM); m.p. = 103–105 °C; ^1^H NMR (500 MHz, CDCl_3_): δ 8.98 (d, *J* = 4.5 Hz, 1H), 8.32 (d, *J* = 8.5 Hz, 1H), 8.17 (d, *J* = 8.5 Hz, 1H), 7.78–7.74 (m, 1H), 7.67–7.63 (m, 1H), 7.59 (d, *J* = 5.0 Hz, 1H), 7.56 (s, 1H), 6.51 (d, *J* = 11.5 Hz, 1H), 5.92–5.85 (m, 1H), 5.78 (d, *J* = 3.0 Hz, 1H), 5.09 (s, 1H), 5.07 (d, *J* = 5.5 Hz, 1H), 4.72 (s, 2H), 4.50 (d, *J* = 4.0 Hz, 1H), 4.25–4.21 (m, 1H), 4.07–3.90 (m, 5H), 3.37–3.32 (m, 1H), 3.22–3.17 (m, 1H), 2.78–2.72 (m, 2H), 2.32 (bs, 1H), 1.81–1.58 (m, 4H), 1.45 (s, 3H), 1.36 (s, 3H), 1.29 (s, 3H), 1.24 (s, 3H), 0.87–0.83 (m, 1H); ^13^C NMR (125 MHz, CDCl_3_): δ 150.2, 148.9, 144.7, 141.4, 141.1, 130.8, 129.8, 127.9, 127.0, 122.4, 121.2, 119.4, 114.8, 111.8, 109.0, 105.2, 82.5, 82.0, 81.1, 72.4, 67.3, 64.5, 58.5, 56.1, 53.4, 41.1, 39.2, 27.8, 27.6, 27.1, 26.9, 26.8, 26.2 and 25.5 ppm. HRMS m/z [M + H]^+^ calculated for C_34_H_44_N_5_O_6_^+^ 618.3287, found 618.3291.

#### 9-Deoxy-9-(4-(1,2-O-isopropylidine-3-O-methyl-α-D-glucofuranos-3-yl)-1H-1,2,3-triazole-1-yl]-epi-cinchonidine (3b)

Light brown solid, yield 67%; R_*f*_ = 0.3 (5% MeOH/DCM); m.p. = 112–114 °C; ^1^H NMR (500 MHz, CDCl_3_): δ 8.99 (d, *J* = 4.5 Hz, 1H), 8.32 (d, *J* = 9.0 Hz, 1H), 8.16 (d, *J* = 8.0 Hz, 1H), 7.78–7.74 (m, 1H), 7.67–7.64 (m, 1H), 7.54 (d, *J* = 4.5 Hz, 1H), 7.51 (s, 1H), 6.57 (d, *J* = 11.5 Hz, 1H), 5.91–5.84 (m, 2H), 5.10–5.06 (m, 2H), 4.77 (d, *J* = 14.0 Hz, 1H), 4.56–4.53 (m, 2H), 4.17 (d, *J* = 3.0 Hz, 1H), 4.04–4.02 (m, 1H), 3.93–3.88 (m, 2H), 3.78–3.75 (m, 1H), 3.63–3.60 (m, 1H), 3.44–3.38 (m, 1H), 3.23–3.19 (m, 1H), 2.77–2.71 (m, 2H), 2.36 (bs, 1H), 1.87–1.82 (m, 1H), 1.78–1.76 (m, 1H), 1.65–1.62 (m, 2H), 1.43 (s, 3H), 1.28 (s, 3H), 0.88–0.83 (m, 1H); ^13^C NMR (125 MHz, CDCl_3_): δ 150.1, 148.9, 144.4, 140.9, 140.5, 130.8, 130.0, 128.2, 126.9, 122.4, 120.6, 119.0, 115.2, 111.9, 105.2, 82.5, 81.7, 80.5, 68.2, 65.0, 62.7, 60.1, 58.3, 56.0, 40.9, 38.9, 27.4, 27.3, 27.2, 26.7 and 26.3 ppm. HRMS m/z [M + H]^+^ calculated for C_31_H_40_N_5_O_6_^+^ 578.2974, found 578.2951.

#### 9-Deoxy-9-(4-(1,2-O-isopropylidine-3-O-methyl-α-D-xylofuranos-3-yl)-1H-1,2,3-triazole-1-yl]-epi-cinchonidine (3c)

Light brown solid, yield 62%; R_*f*_ = 0.3 (5% MeOH/DCM); m.p. = 83–85 °C; ^1^H NMR (500 MHz, CDCl_3_): δ 8.95 (d, *J* = 3.5 Hz, 1H), 8.33 (d, *J* = 8.5 Hz, 1H), 8.13 (d, *J* = 8.5 Hz, 1H), 7.73–7.70 (m, 1H), 7.63–7.54 (m, 3H), 6.55 (d, *J* = 11.5 Hz, 1H), 5.89–5.83 (m, 2H), 5.07–5.03 (m, 2H), 4.71 (d, *J* = 13.5 Hz, 1H), 4.54–4.51 (m, 2H), 4.17–4.16 (m, 1H), 4.09–4.07 (m, 1H), 3.93–3.87 (m, 1H), 3.70–3.63 (m, 2H), 3.37–3.32 (m, 1H), 3.19–3.14 (m, 1H), 2.73–2.68 (m, 2H), 2.31 (bs, 1H), 1.84–1.80 (m, 1H), 1.73 (s, 1H), 1.59 (s, 2H), 1.41 (s, 3H), 1.24 (s, 3H), 2.85–0.81 (m, 1H); ^13^C NMR (125 MHz, CDCl_3_): δ 149.9, 148.6, 144.5, 140.8, 140.5, 130.5, 129.8, 127.9, 126.7, 122.3, 120.7, 118.9, 114.9, 111.6, 104.8, 82.6, 81.5, 79.7, 63.2, 59.7, 58.7, 58.2, 55.6, 40.6, 38.7, 29.5, 27.3, 27.0, 26.6 and 26.1 ppm. HRMS m/z [M + H]^+^ calculated for C_30_H_38_N_5_O_5_^+^ 548.2868, found 548.2869.

#### 9-Deoxy-9-(4-(1,2-O-isopropylidine-5-O-methyl-3-O-propyl-α-D-xylofuranos-5-yl)-1H-1,2,3-triazole-1-yl]-epi-cinchonidine (3d)

Light brown solid, yield 65%; R_*f*_ = 0.4 (5% MeOH/DCM); m.p. = 133–135 °C; ^1^H NMR (500 MHz, CDCl_3_): δ 8.97 (d, *J* = 4.5 Hz, 1H), 8.33 (d, *J* = 7.5 Hz, 1H), 8.16 (d, *J* = 8.5 Hz, 1H), 7.77–7.74 (m, 1H), 7.67–7.58 (m, 3H), 6.52 (d, *J* = 11.5 Hz, 1H), 5.93–5.86 (m, 2H), 5.08 (d, *J* = 14.5 Hz, 2H), 4.62 (s, 2H), 4.50 (d, *J* = 3.5 Hz, 1H), 4.34–4.31 (m, 1H), 3.96–3.90 (m, 1H), 3.75–3.66 (m, 3H), 3.51–3.46 (m, 1H), 3.38–3.34 (m, 1H), 3.31–3.26 (m, 1H), 3.19 (dd, *J* = 10.0, 4.0 Hz, 1H), 2.78–2.72 (m, 2H), 2.32 (bs, 1H), 1.97 (bs, 1H), 1.80–1.74 (m, 2H), 1.62–1.57 (m, 1H), 1.50–1.47 (m, 2H), 1.45 (s, 3H), 1.29 (s, 3H), 0.86–0.81 (m, 4H).; ^13^C NMR (125 MHz, CDCl_3_): δ 150.1, 148.8, 144.9, 141.4, 141.1, 130.7, 129.8, 127.9, 127.0, 122.5, 121.4, 119.4, 114.8, 111.6, 105.1, 82.5, 82.3, 79.3, 72.0, 68.4, 65.3, 59.8, 58.4, 56.0, 41.0, 39.2, 27.7, 27.6, 27.1, 26.8, 26.3, 22.9 and 10.5 ppm. HRMS m/z [M + H]^+^ calculated for C_33_H_44_N_5_O_5_^+^ 590.3337, found 590.3345.

#### 9-Deoxy-9-(4-(2,3,4,6-tetra-O-benzyl-1-O-methyl-α-D-glucopyranos-1-yl)-1H-1,2,3-triazole-1-yl]-epi-cinchonidine (3e)

Light brown solid, yield 71%; R_*f*_ = 0.5 (5% MeOH/DCM); m.p. = 60–62 °C; ^1^H NMR (500 MHz, CDCl_3_): δ 8.77 (d, *J* = 4.5 Hz, 1H), 8.19 (d, *J* = 8.0 Hz, 1H), 8.06 (d, *J* = 8.5 Hz, 1H), 7.66–7.63 (m, 1H), 7.55–7.52 (m, 1H), 7.46 (s, 1H), 7.41–7.38 (m, 1H), 7.30–7.17 (m, 18H), 7.08–7.06 (m, 2H), 6.34 (d, *J* = 11.5 Hz, 1H), 5.84–5.77 (m, 1H), 5.02–4.97 (m, 2H), 4.90–4.61 (m, 6H), 4.51–4.41 (m, 3H), 4.31 (d, *J* = 7.5 Hz, 1H), 3.69–3.48 (m, 4H), 3.37–3.34 (m, 1H), 3.26–3.16 (m, 2H), 3.09–3.04 (m, 1H), 2.65–2.60 (m, 2H), 2.21 (bs, 1H), 1.88 (bs, 1H), 1.64 (s, 2H), 1.49–1.48 (m, 2H), 1.18 (s, 1H), 0.72–0.69 (m, 1H).; ^13^C NMR (125 MHz, CDCl_3_): δ 150.2, 144.7, 141.4, 141.0, 138.7, 138.6, 138.1, 130.8, 129.8, 128.5, 128.4, 128.0, 127.9, 127.8, 127.7, 127.7, 126.0, 122.5, 121.7, 119.4, 114.8, 102.2, 84.7, 82.2, 77.8, 75.7, 75.1, 75.0, 74.7, 74.6, 73.5, 68.9, 63.2, 58.3, 56.1, 41.0, 39.3, 27.8, 27.6 and 27.1 ppm. HRMS m/z [M + H]^+^ calculated for C_56_H_60_N_5_O_6_^+^ 898.4539, found 618.4543.

#### 9-Deoxy-9-(4-(2,3;5,6-di-O-isopropylidine-1-O-methyl-α-D-mannofuranos-1-yl)-1H-1,2,3-triazole-1-yl]-epi-cinchonidine (3f)

Light brown solid, yield 54%; R_*f*_ = 0.2 (5% MeOH/DCM); m.p. = 150–152 °C; ^1^H NMR (500 MHz, CDCl_3_): δ 8.97 (d, *J* = 5.0 Hz, 1H), 8.31 (d, *J* = 8.5 Hz, 1H), 8.17 (d, *J* = 8.5 Hz, 1H), 7.77–7.73 (m, 1H), 7.67–7.58 (m, 3H), 6.51 (d, *J* = 11.5 Hz, 1H), 5.93–5.85 (m, 1H), 5.09–5.06 (m, 2H), 4.88–4.83 (m, 2H), 4.77 (d, *J* = 12.5 Hz, 1H), 4.67–4.65 (m, 1H), 4.53–4.51 (m, 1H), 4.38–4.37 (m, 1H), 4.01 (d, *J* = 6.0 Hz, 2H), 3.56–3.54 (m, 1H), 3.39–3.33 (m, 1H), 3.20–3.15 (m, 1H), 2.78–2.74 (m, 2H), 2.32 (bs, 1H), 1.81–1.74 (m, 2H), 1.61–1.59 (m, 2H), 1.43 (s, 3H), 1.42 (s, 3H), 1.35 (s, 3H), 1.30 (s, 3H), 0.89–0.82 (m, 1H).; ^13^C NMR (125 MHz, CDCl_3_): δ 149.9, 148.6, 144.0, 141.2, 140.9, 130.5, 129.6, 127.7, 126.7, 122.2, 122.0, 114.6, 113.4, 109.0, 101.7, 79.4, 78.9, 73.0, 66.6, 63.2, 59.7, 58.2, 55.8, 53.3, 40.8, 39.0, 29.5, 27.5, 27.4, 26.9, 26.8, 25.4, 25.1 and 24.8 ppm. HRMS m/z [M + H]^+^ calculated for C_34_H_44_N_5_O_6_^+^ 618.3287, found 618.3286.

#### 9-Deoxy-9-(4-(2,3;5,6-di-O-isopropylidine-1-O-methyl-β-D-mannofuranos-1-yl)-1H-1,2,3-triazole-1-yl]-epi-cinchonidine (3g)

Light brown solid, yield 59%; R_*f*_ = 0.3 (5% MeOH/DCM); m.p. = 102–104 °C; ^1^H NMR (500 MHz, CDCl_3_): δ 8.95 (d, *J* = 4.5 Hz, 1H), 8.29 (d, *J* = 8.5 Hz, 1H), 8.15 (d, *J* = 7.5 Hz, 1H), 7.76–7.72 (m, 1H), 7.65–7.62 (m, 2H), 7.57 (d, *J* = 3.5 Hz, 1H), 6.49 (d, *J* = 11.0 Hz, 1H), 5.91–5.84 (m, 1H), 5.08–5.04 (m, 2H), 4.86–4.81 (m, 2H), 4.76–4.72 (m, 1H), 4.65–4.63 (m, 1H), 4.51–4.49 (m, 1H), 4.37–4.33 (m, 1H), 4.10–4.07 (m, 1H), 3.99 (d, *J* = 4.5 Hz, 2H), 3.54–3.52 (m, 1H), 3.37–3.31 (m, 1H), 3.18–3.13 (m, 1H), 2.76–2.70 (m, 2H), 2.31 (bs, 1H), 1.77–1.73 (m, 2H), 1.60–1.57 (m, 2H), 1.41 (s, 3H), 1.40 (s, 3H), 1.38 (s, 3H), 1.28 (s, 3H), 0.87–0.85 (m, 1H).; ^13^C NMR (125 MHz, CDCl_3_): δ 149.8, 148.5, 143.9, 141.0, 140.8, 130.4, 129.5, 127.6, 126.6, 122.1, 121.9, 114.6, 113.3, 109.0, 101.6, 79.2, 78.8, 72.9, 66.5, 63.1, 58.1, 55.6, 53.2, 40.7, 38.9, 31.3, 27.4, 27.3, 26.8, 26.7, 25.3, 25.0, 24.7, and 22.4. ppm. HRMS m/z [M + H]^+^ calculated for C_34_H_44_N_5_O_6_^+^ 618.3287, found 618.3283.

#### 9-Deoxy-9-(4-(2,3-O-isopropylidine-1-O-methyl-β-D-mannofuranos-1-yl)-1H-1,2,3-triazole-1-yl]-epi-cinchonidine (**3h**)

Light brown solid, yield 60%; R_*f*_ = 0.2 (5% MeOH/DCM); ^1^H NMR (500 MHz, CDCl_3_): δ 8.95 (d, *J* = 4.0 Hz, 1H), 8.32 (d, *J* = 9.0 Hz, 1H), 8.16 (d, *J* = 8.5 Hz, 1H), 7.76–7.73 (m, 1H), 7.67–7.61 (m, 3H), 6.52 (d, *J* = 10.5 Hz, 1H), 5.91–5.81 (m, 1H), 5.08–5.05 (m, 3H), 4.78–4.76 (m, 1H), 4.64 (d, *J* = 12.5 Hz, 1H), 4.57–4.52 (m, 2H), 4.04–3.93 (m, 3H), 3.78–3.70 (m, 2H), 3.49 (bs, 1H), 3.38–3.34 (m, 1H), 3.20–3.15 (m, 1H), 2.80–2.72 (m, 2H), 2.31 (bs, 1H), 1.77–1.73 (m, 2H), 1.60 (bs, 2H), 1.42 (s, 3H), 1.28 (s, 3H), 0.83–0.79 (m, 1H).; ^13^C NMR (125 MHz, CDCl_3_): δ 150.0, 148.5, 144.1, 141.1, 130.5, 129.7, 127.8, 126.7, 122.2, 121.9, 119.4, 114.7, 113.9, 112.3, 106.2, 84.6, 79.7, 78.7, 69.5, 63.4, 60.4, 58.0, 55.6, 53.3, 40.8, 38.9, 27.5, 27.4, 26.8, 25.8 and 24.5 ppm. HRMS m/z [M + H]^+^ calculated for C_31_H_40_N_5_O_6_^+^ 578.2974, found 578.2973.

#### 9-Deoxy-9-(4-(2,3-O-isopropylidine-1-O-methyl-β-D-lyxofuranos-1-yl)-1H-1,2,3-triazole-1-yl]-epi-cinchonidine (3i)

Light brown solid, yield 48%; R_*f*_ = 0.2 (5% MeOH/DCM); m.p. = 70–74 °C; ^1^H NMR (500 MHz, CDCl_3_): δ 8.91 (d, *J* = 5.0 Hz, 1H), 8.28 (d, *J* = 8.5 Hz, 1H), 8.10 (d, *J* = 8.5 Hz, 1H), 7.69–7.66 (m, 1H), 7.61–7.55 (m, 3H), 6.49 (d, *J* = 11.5 Hz, 1H), 5.85–5.78 (m, 1H), 5.04–5.49 (m, 3H), 4.65–4.62 (m, 2H), 4.52–4.47 (m, 2H), 3.97–3.88 (m, 2H), 2.83–3.75 (m, 2H), 3.37–3.29 (m, 1H), 3.14–3.09 (m, 1H), 2.72–2.66 (m, 2H), 2.26 (bs, 1H), 1.74–1.66 (m, 2H), 1.54–1.53 (m, 2H),1.35 (s, 3H), 1.18 (s, 3H), 0.78–0.74 (m, 1H).; ^13^C NMR (125 MHz, CDCl_3_): δ 150.1, 148.6, 144.2, 141.3, 141.2, 130.6, 129.8, 127.9, 126.9, 122.5, 122.0, 119.5, 114.9, 112.6, 105.9, 85.1, 80.1, 80.0, 60.7, 58.3, 55.8, 53.5, 40.9, 39.1, 31.9, 29.9, 27.6, 27.0, 25.9, and 24.6 ppm. HRMS m/z [M + H]^+^ calculated for C_30_H_38_N_5_O_5_^+^ 548.2868, found 548.2856.

#### 9-Deoxy-9-(4-(1,2;3,4-di-O-isopropylidine-6-O-methyl-α-D-galactopyranos-6-yl)-1H-1,2,3-triazole-1-yl]-epi-cinchonidine (3j)

Light brown solid, yield 67%; R_*f*_ = 0.4 (5% MeOH/DCM); m.p. = 130–132 °C; ^1^H NMR (500 MHz, CDCl_3_): δ 8.97 (s, 1H), 8.34 (d, *J* = 8.0 Hz, 1H), 8.17 (d, *J* = 8.5 Hz, 1H), 7.77–7.74 (m, 1H), 7.67–7.63 (m, 2H), 7.58 (s, 1H), 6.52 (d, *J* = 11.0 Hz, 1H), 4.92–5.85 (m, 1H), 5.50 (d, *J* = 5.0 Hz, 1H), 5.09–5.06 (m, 2H), 4.64 (d, *J* = 1.5 Hz, 2H), 4.56–4.54 (m, 1H), 4.28–4.27 (m, 1H), 4.18–4.16 (m, 1H), 3.97–3.94 (m, 2H), 3.68–3.59 (m, 2H), 3.37–3.33 (m, 1H), 3.21–3.17 (m, 1H), 2.78–2.75 (m, 2H), 2.32 (bs, 1H), 1.79–1.74 (m, 2H), 1.61–1.59 (m, 2H), 1.48 (s, 3H), 1.36 (s, 3H), 1.30 (s, 3H), 1.28 (s, 3H), 0.86–0.82 (m, 1H).; ^13^C NMR (125 MHz, CDCl_3_): δ 149.9, 148.8, 145.2, 141.4, 141.1, 130.8, 129.8, 127.7, 127.2, 122.5, 121.4, 119.4, 114.8, 109.3, 108.6, 96.4, 71.1, 70.6, 70.5, 69.5, 66.6, 65.1, 58.4, 56.0, 41.1, 39.2, 27.8, 27.6, 27.1, 26.1, 26.0, 24.9 and 24.5 ppm. HRMS m/z [M + H]^+^ calculated for C_34_H_44_N_5_O_6_^+^ 618.3287, found 618.3297.

## Supplementary information

Supplementary information.
