# Supplementary material for: Click Inspired Synthesis of Novel Cinchonidine Glycoconjugates as Promising Plasmepsin Inhibitors
Source: Sci Rep. 2020 Feb 27;10:3586. doi: 10.1038/s41598-020-59477-3 (PMC7046651; doi:10.1038/s41598-020-59477-3)
Supplement: Supplementary file 1 — Supplementary information. [file 41598_2020_59477_MOESM1_ESM.doc]

**Click Inspired Synthesis of Novel Cinchonidine** Glycoconjugates as Promising Plasmepsin Inhibitors

Nidhi Mishra^1^, Anand K. Agrahari^1^, Priyanka Bose^1^, Sumit K. Singh^1^, Anoop S. Singh^1^, and Vinod K. Tiwari*^1^

^1^Department of Chemistry, Institute of Science, Banaras Hindu University, Varanasi-221005, India

*E-mail: [tiwari_chem@yahoo.co.in](mailto:tiwari_chem@yahoo.co.in), [vinod.tiwari@bhu.ac.in](mailto:vinod.tiwari@bhu.ac.in)

| **Table of Contents** | | |
| --- | --- | --- |
| 1 | ^1^H and ^13^C NMR Spectra of azido cinchonidine **1** | S2-S3 |
| 2 | ^1^H and ^13^C NMR Spectra of *O-*Propargyl ether derivatives of sugars **2a-j** | S4-S17 |
| 3 | ^1^H and ^13^C NMR Spectra of Cinchonidine glycoconjugates **3a-j** | S18-S37 |
| 4 | Optimized structures and IR spectra of cinchonidine glycoconjugate **3a-j** fragments generated by Gaussian method β3LYP/6-311g(d,p). | S38-S43 |
| 5 | Pictures of fragment molecular docking **3a-j** using AutoDock and AutoDock Vina | S44-S54 |
| 6 | Pictures of whole molecular docking **3a-j** using AutoDock Vina**.** | S55-S58 |
| 7 | The characteristic chemical properties computed using optimized structures which directly influences the pharmacokinetics properties of the developed cinchona glycoconjugates | S59-S60 |
|  |  |  |

1. ^1^H and ^13^C NMR Spectra of Azido cinchonidine **1**

**Spectra 1.** ^1^H NMR (500 MHz, CDCl_3_) of compound **1**

**Spectra 2.** ^13^C NMR (125 MHz, CDCl_3_) of compound **1**

1. ^1^H and ^13^C NMR Spectra of *O*-Propargyl ether derivatives of sugars (**2a-j**)

**Spectra 3.** ^1^H NMR (500 MHz, CDCl_3_) of 1,2-*O*-isopropylidene-3-*O*-propyl-α-D-xylofuranose

**Spectra 4.** ^13^CNMR (125 MHz, CDCl_3_) of 1,2-*O*-isopropylidene-3-*O*-propyl-α-D-xylofuranose

**Spectra 5.** ^1^HNMR (500 MHz, CDCl_3_) of compound **2d**

**Spectra 6.** ^13^CNMR (125 MHz, CDCl_3_) of compound **2d**

**Spectra 7.** ^1^H NMR (500 MHz, CDCl_3_) of compound **2f**

**Spectra 8.** ^13^C NMR (125 MHz, CDCl_3_) of compound **2f**

**Spectra 9.** ^1^H NMR (500 MHz, CDCl_3_) of compound **2g**

**Spectra 10.** ^13^C NMR (125 MHz, CDCl_3_) of compound **2g**

**Spectra 11.** ^1^H NMR (500 MHz, CDCl_3_) of compound **2h**

**Spectra 12.** ^13^C NMR (125 MHz, CDCl_3_) of compound **2h**

**Spectra 13.** ^1^H NMR (500 MHz, CDCl_3_) of *2,3-O-isopropylidene-1-O-(2-propyn-1-yl)-α-D-lyxose-pentadialdo-1,4-furanose*

**Spectra 14.** ^13^C NMR (125 MHz, CDCl_3_) of *2,3-O-isopropylidene-1-O-(2-propyn-1-yl)-α-D-lyxose-pentadialdo-1,4-furanose*

**Spectra 15.** ^1^H NMR (500 MHz, CDCl_3_) of compound **2i**

**Spectra 16.** ^13^C NMR (125 MHz, CDCl_3_) of compound **2i**

1. ^1^H and ^13^C NMR Spectra of Cinchonidine glycoconjugates

**Spectra 17.** ^1^H NMR (500 MHz, CDCl_3_) of compound **3a**

**Spectra 18.** ^13^C NMR (125 MHz, CDCl_3_) of compound **3a**

**Spectra 19.** ^1^H NMR (500 MHz, CDCl_3_) of compound **3b**

**Spectra 20.** ^13^C NMR (125 MHz, CDCl_3_) of compound **3b**

**Spectra 21.** ^1^H NMR (500 MHz, CDCl_3_) of compound **3c**

**Spectra 22.** ^13^C NMR (125 MHz, CDCl_3_) of compound **3c**

**Spectra 23.** ^1^H NMR (500 MHz, CDCl_3_) of compound **3d**

**Spectra 24.** ^13^C NMR (125 MHz, CDCl_3_) of compound **3d**

**Spectra 25.** ^1^H NMR (500 MHz, CDCl_3_) of compound **3e**

**Spectra 26.** ^13^C NMR (125 MHz, CDCl_3_) of compound **3e**

**Spectra 27.** ^1^H NMR (500 MHz, CDCl_3_) spectra of compound **3f**

**Spectra 28.** ^13^C NMR (125 MHz, CDCl_3_) spectra of compound **3f**

**Spectra 29.** ^1^H NMR (500 MHz, CDCl_3_) spectra of compound **3g**

**Spectra 30.** ^13^C NMR (125 MHz, CDCl_3_) spectra of compound **3g**

**Spectra 31.** ^1^H NMR (500 MHz, CDCl_3_) spectra of compound **3h**

**Spectra 32.** ^13^C NMR (125 MHz, CDCl_3_) spectra of compound **3h**

**Spectra 33.** ^1^H NMR (500 MHz, CDCl_3_) spectra of compound **3i**

**Spectra 34.** ^13^C NMR (125 MHz, CDCl_3_) spectra of compound **3i**

**Spectra 35.** ^1^H NMR (500 MHz, CDCl_3_) spectra of compound **3j**

**Spectra 36.** ^13^C NMR (125 MHz, CDCl_3_) spectra of compound **3j**

1. **Table 1.** Optimized structures and IR spectra of cinchonidine glycoconjugate fragments generated by Gaussian method β3LYP/6-311g(d,p).

| **Optimized structure** | **IR Spectra** |
| --- | --- |
| 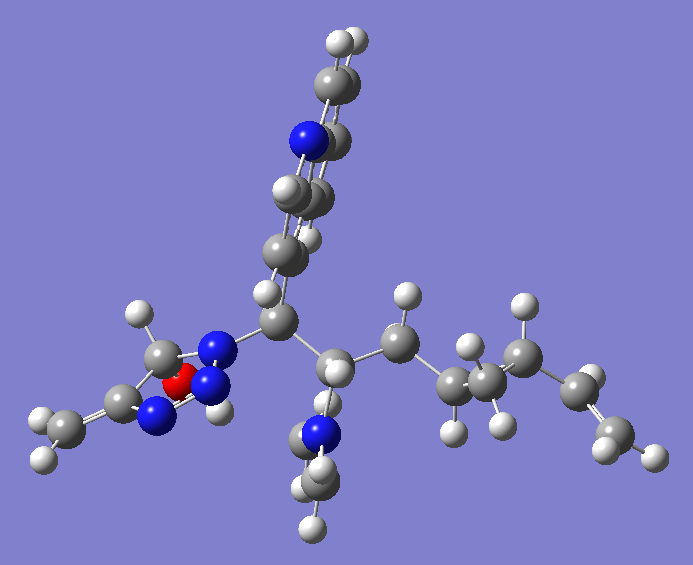  **cm** | 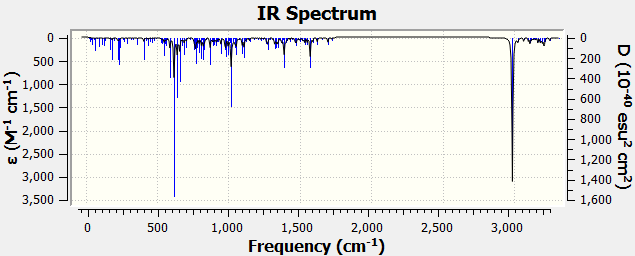  **cm** |
| 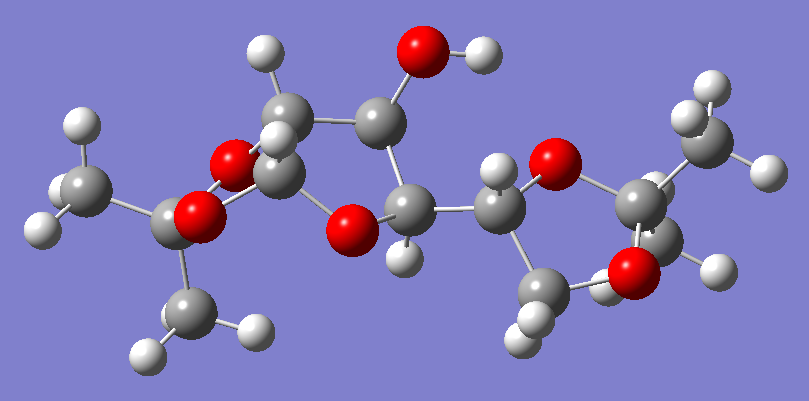  **3a** | 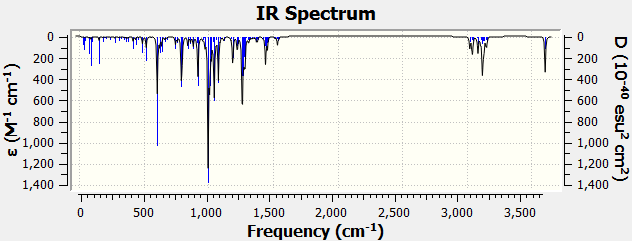  **3a** |
| 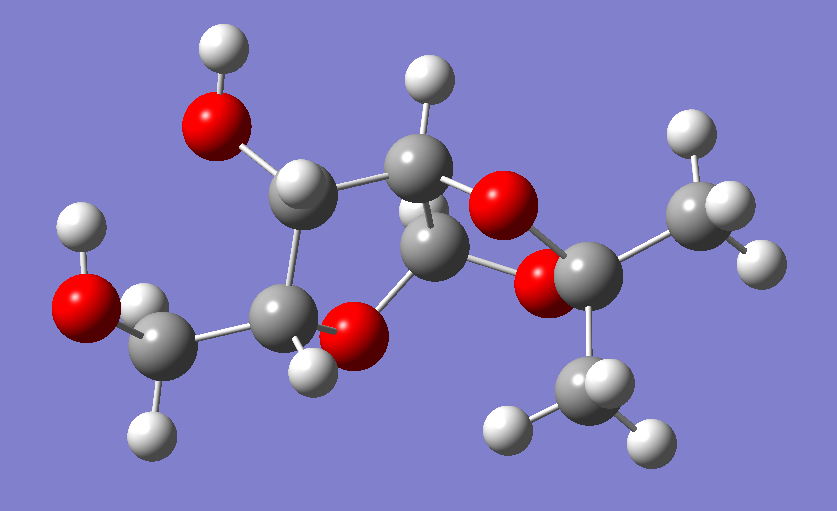  **3b** | 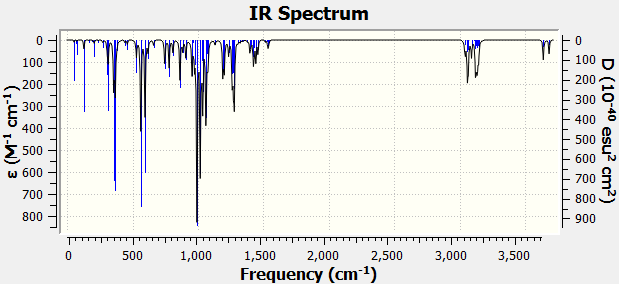  **3b** |
| 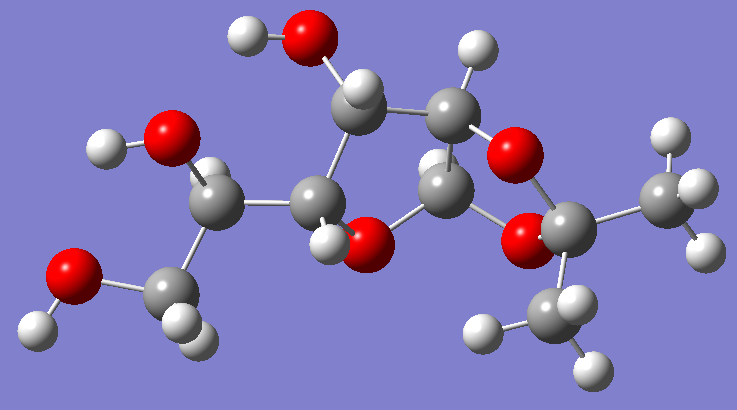  **3c** | 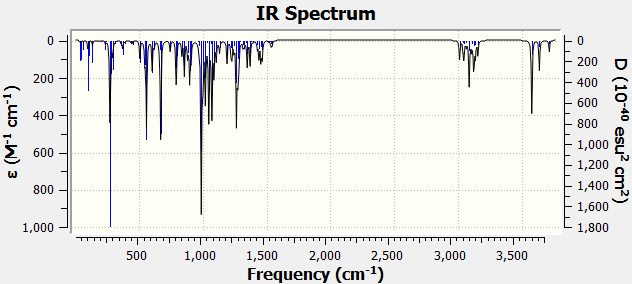  **3c** |
| 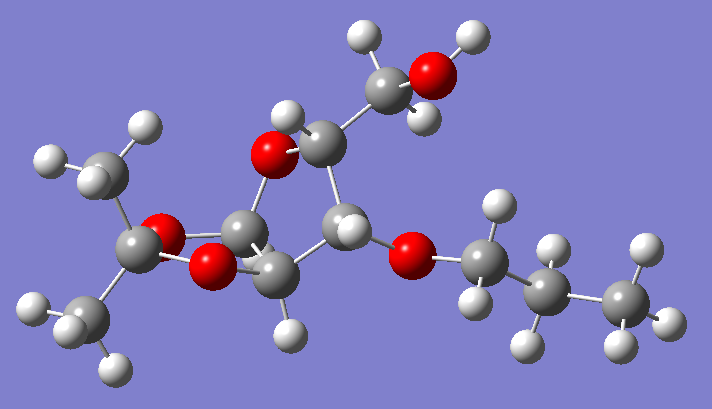  **3d** | 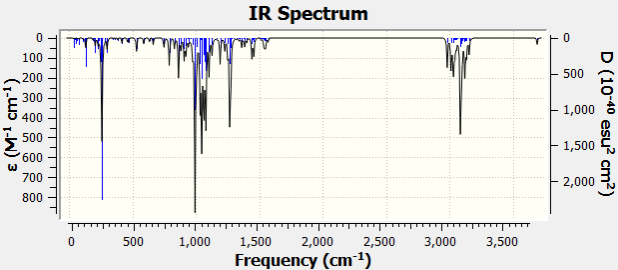  **3d** |
| 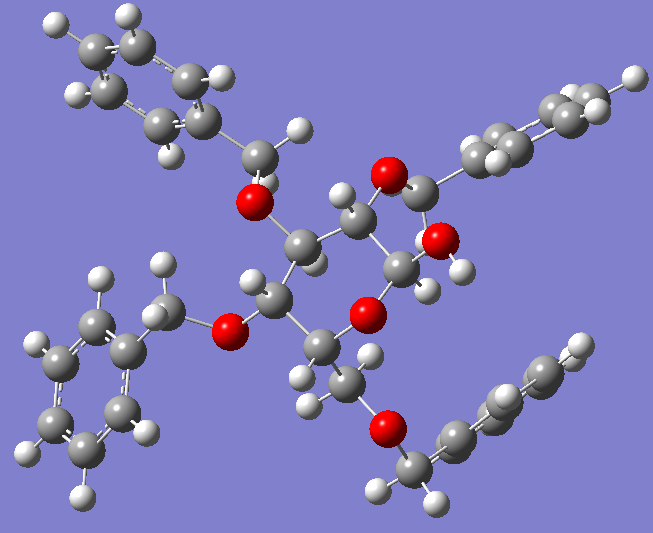  **3e** | 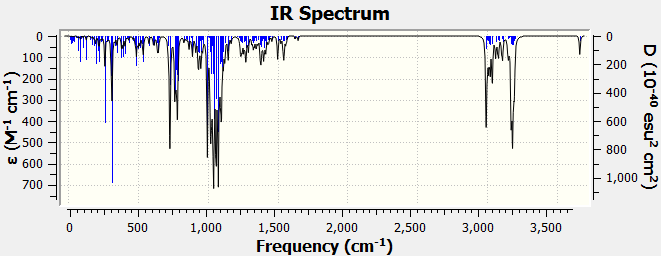  **3e** |
| 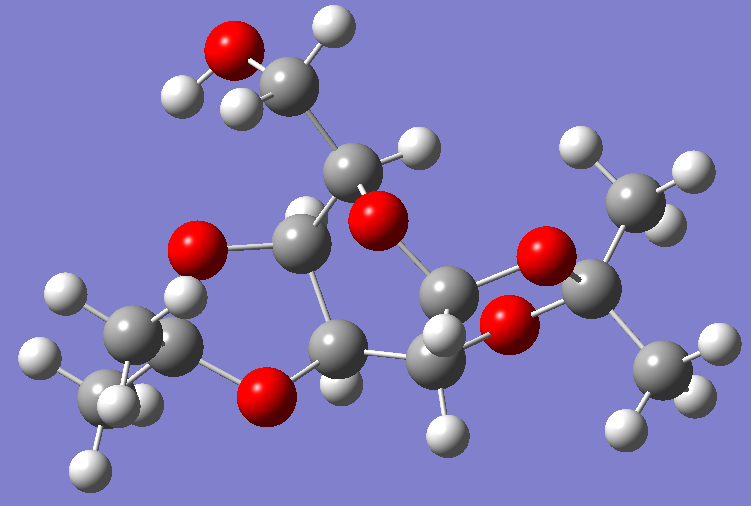  **3f** | 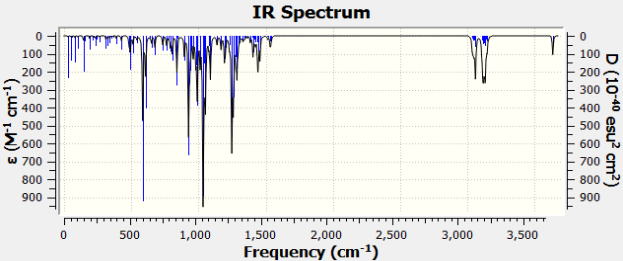  **3f** |
| 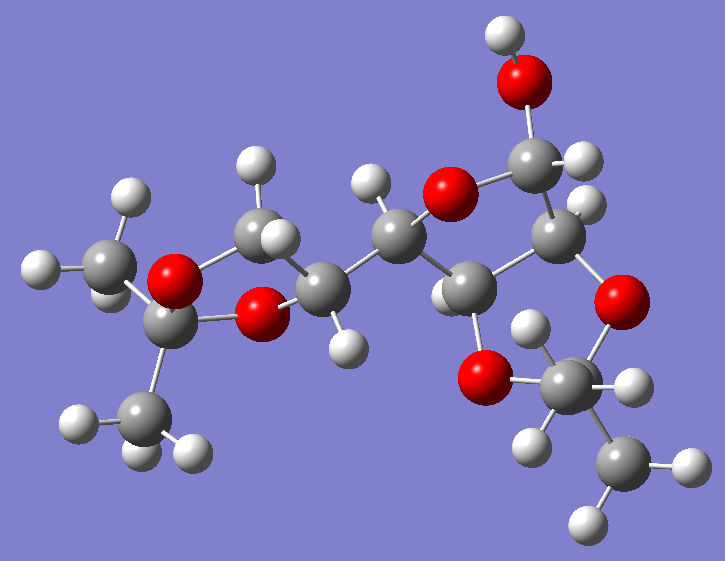  **3g** | 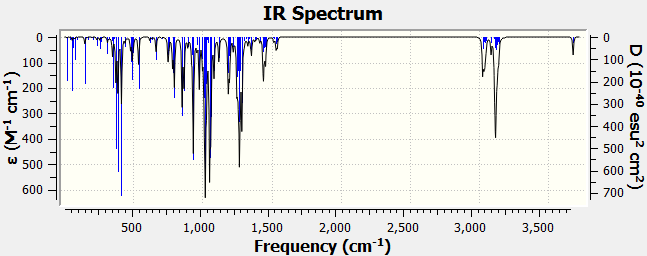  **3g** |
| 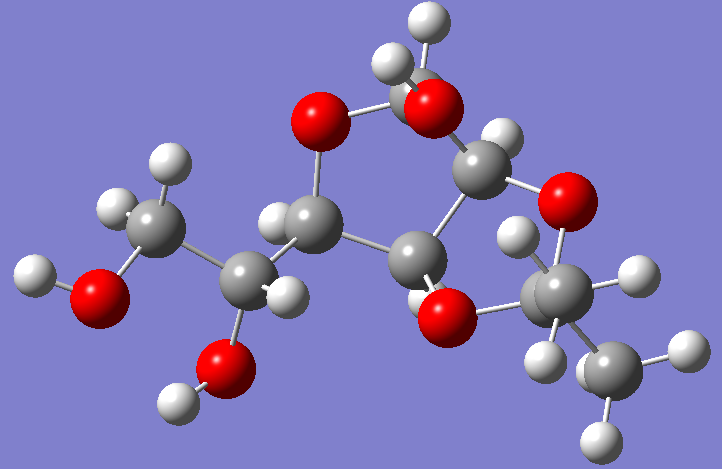  **3h** | 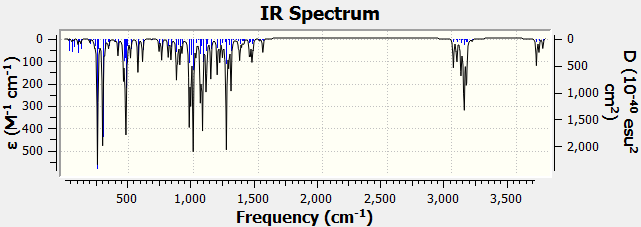  **3h** |
| 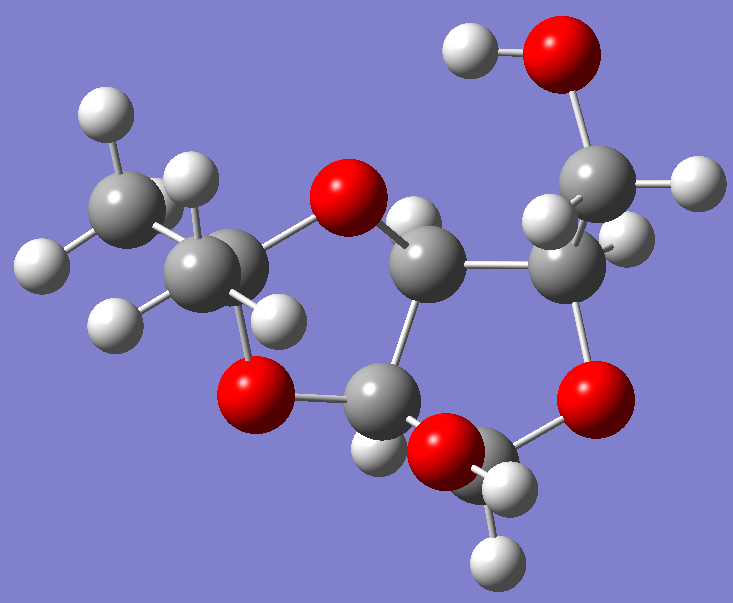  **3i** | 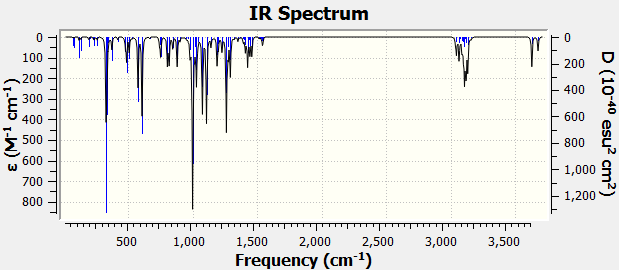  **3i** |
| 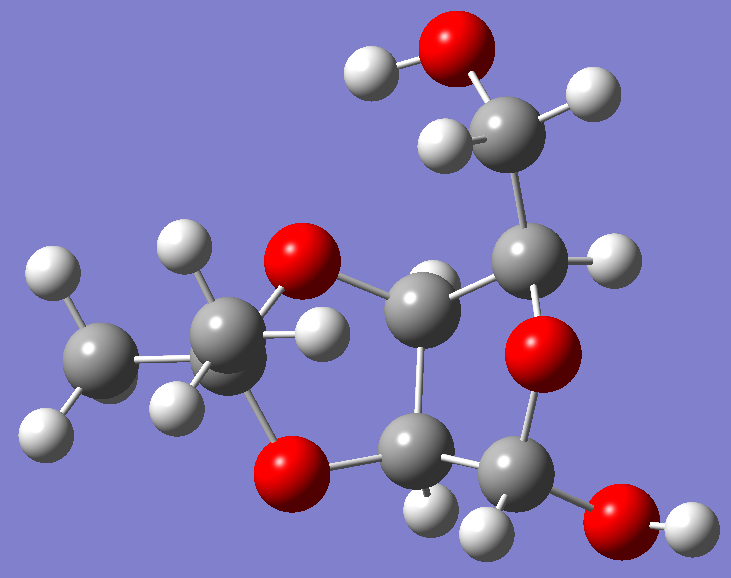  **3j** | 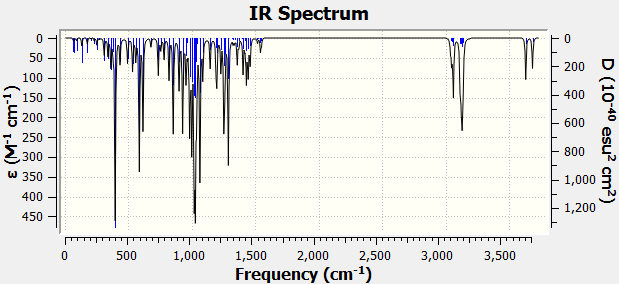  **3j** |

**Table 2.** Optimized structures and IR spectra of cinchonidine glycoconjugates generated by Gaussian method β3LYP/6-311g(d,p).

| Optimized Structure | | IR Spectra |
| --- | --- | --- |
| 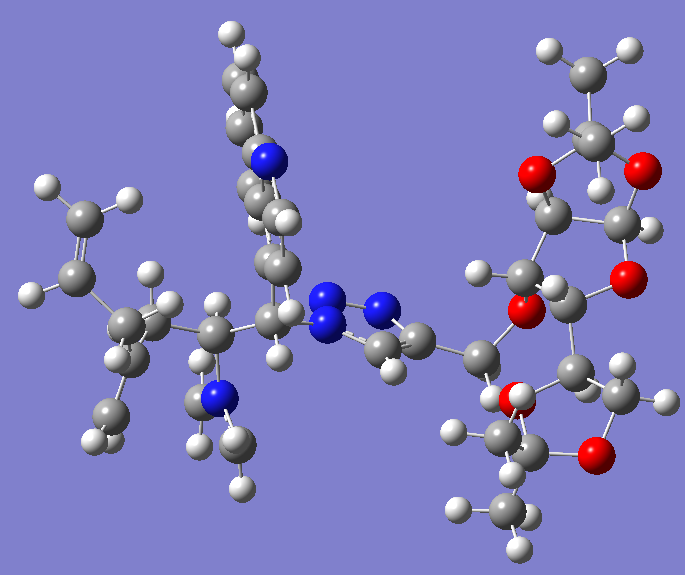  **3a** | | 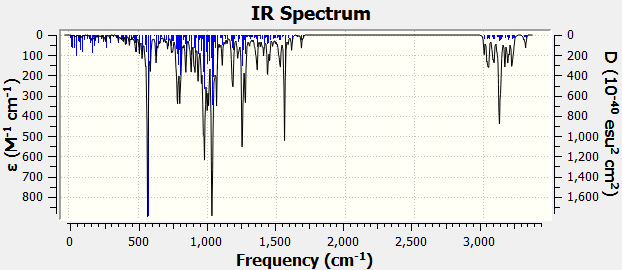  **3a** |
| 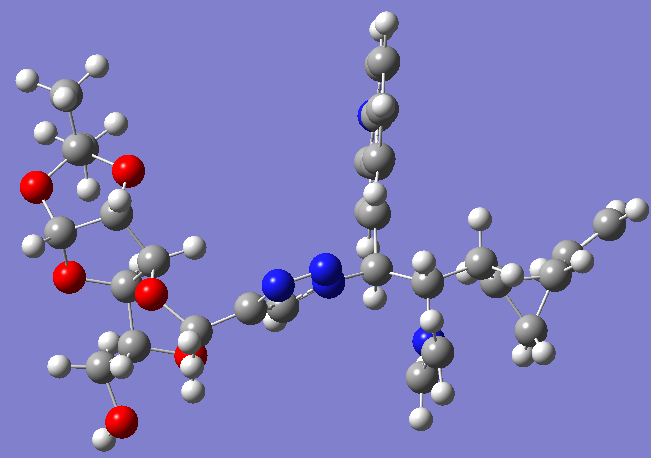  **3b** | | 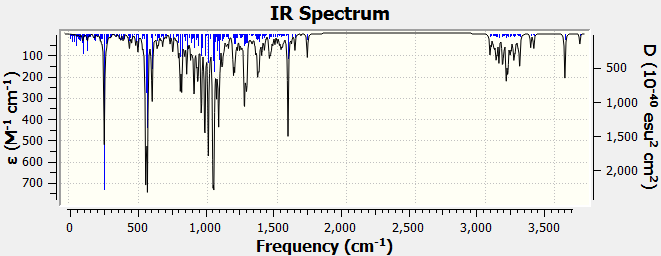  **3b** |
| **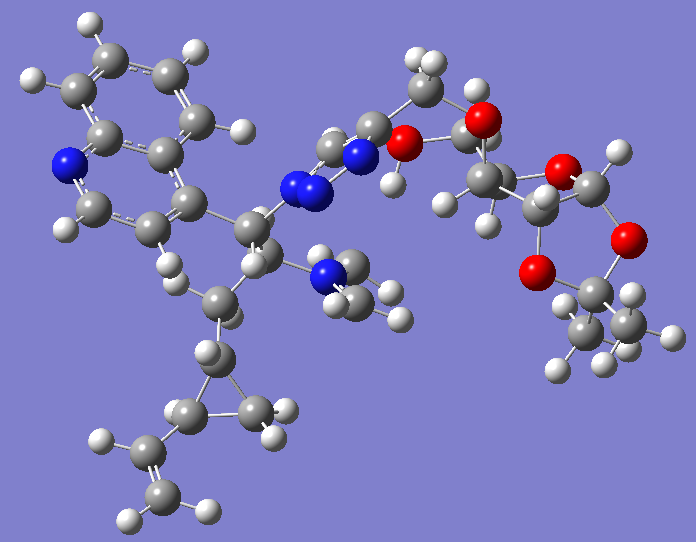**  **3c** | | 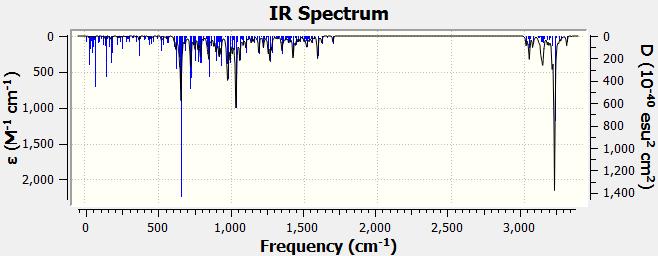  **3c** |
| **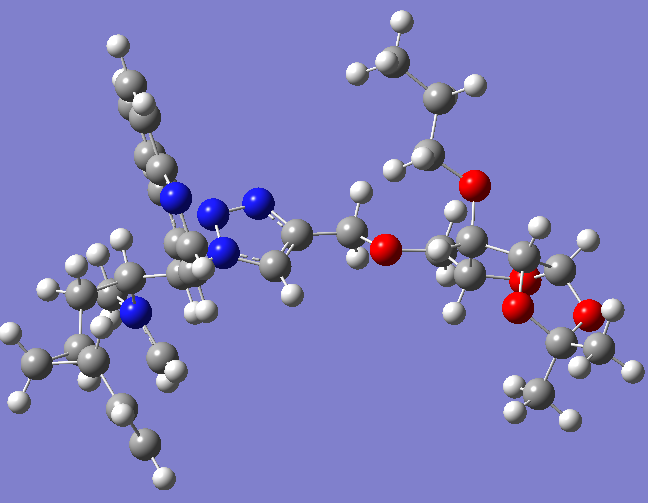**  **3d** | | 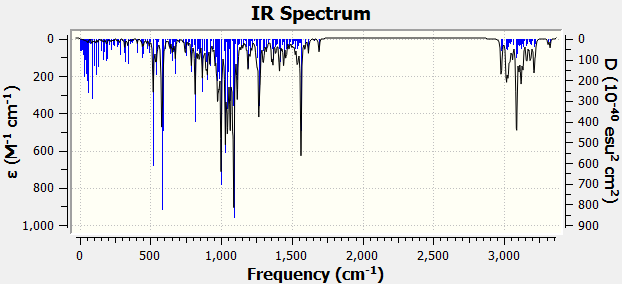  **3d** |
| 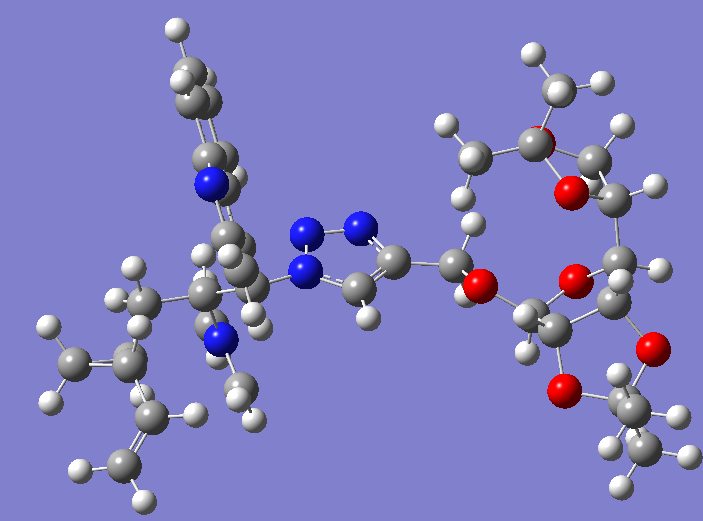  **3f** | | 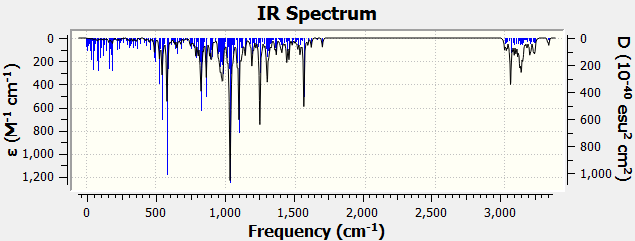  **3f** |
| 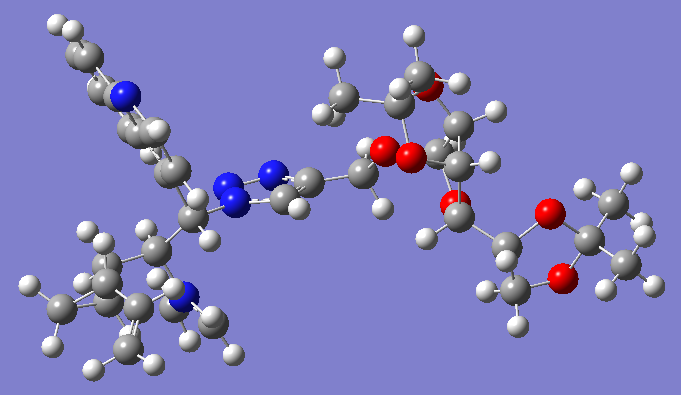  **3g** | | 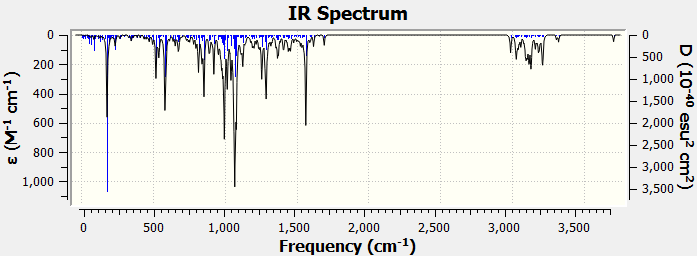  **3g** |
| 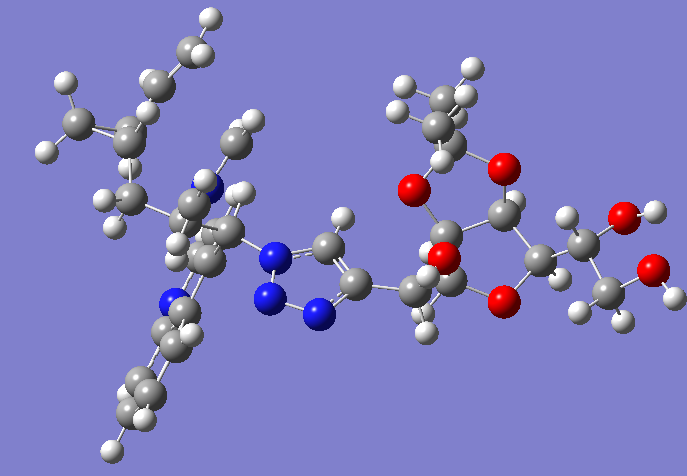  **3h** | | 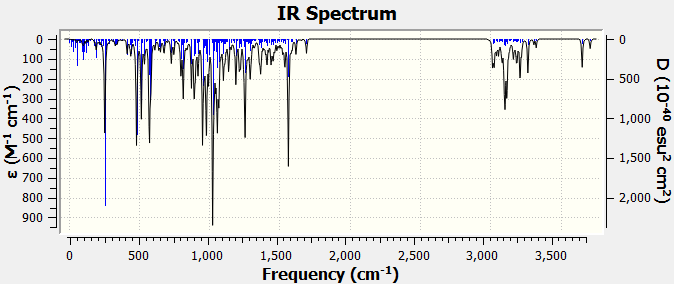  **3h** |
| 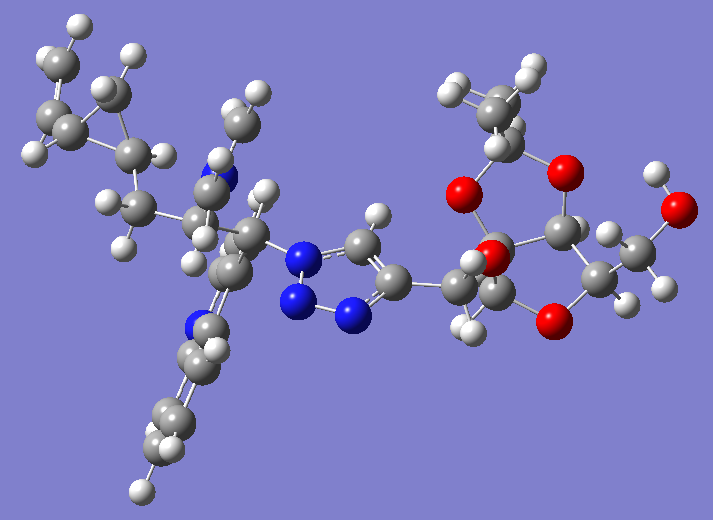  **3i** | | 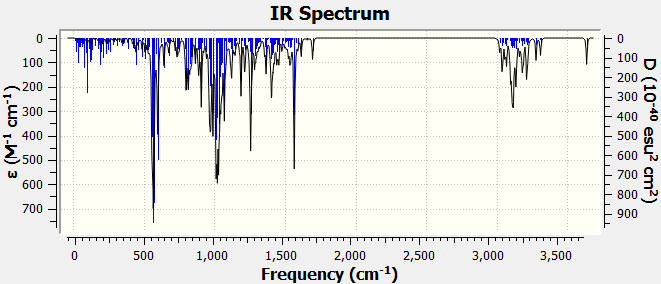  **3i** |
| 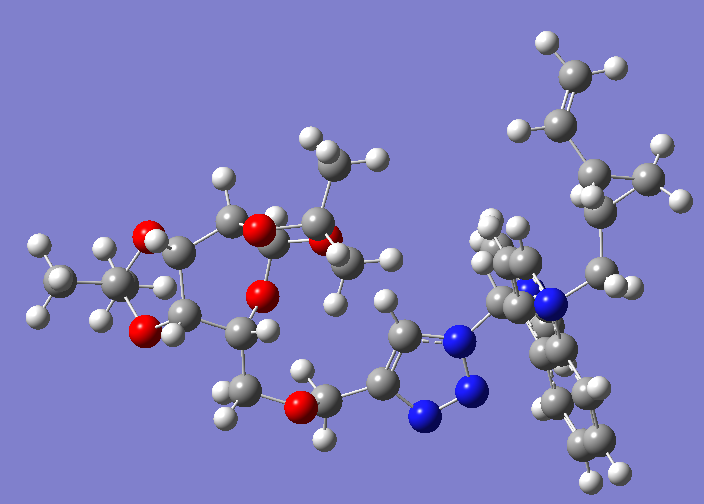  **3j** | 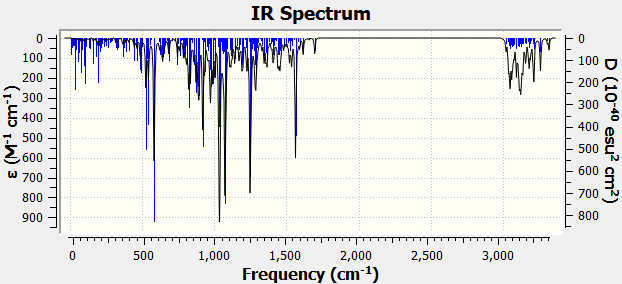  **3j** | |

|  | 1. **Pictures of Fragment Molecular Docking using AutoDock** |
| --- | --- |


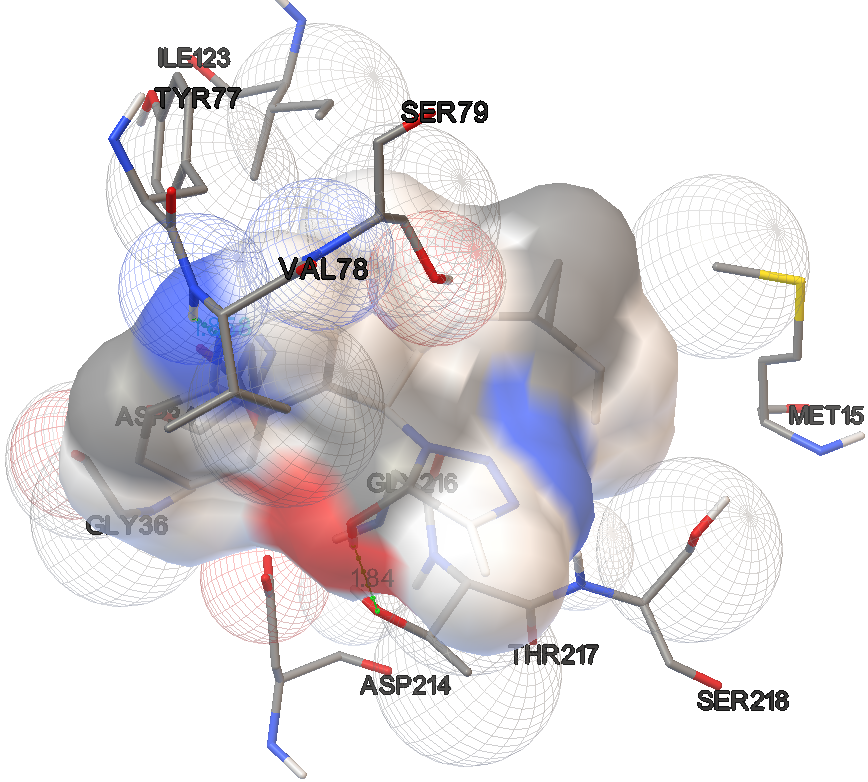


**Figure S1.** Molecular docking picture of central molecule (Cm) fragment (AutoDock).

**
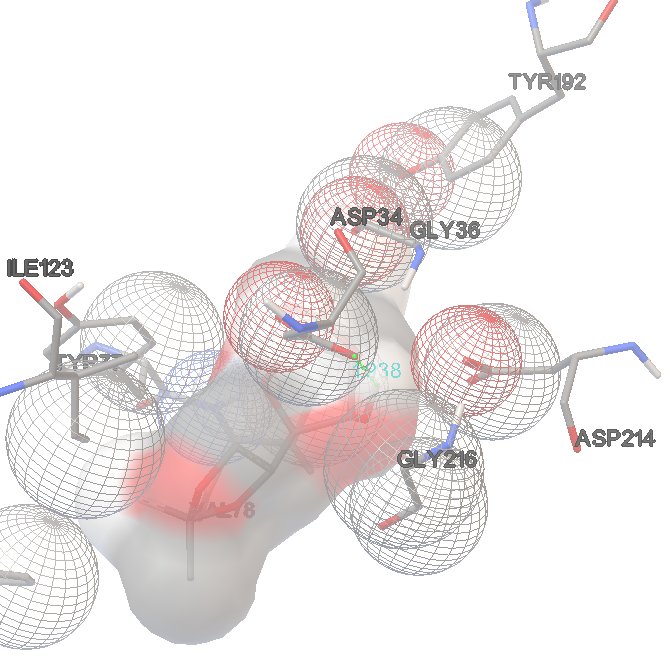
**

**Figure S2.** Molecular docking picture of fragment **3a** (AutoDock).

**
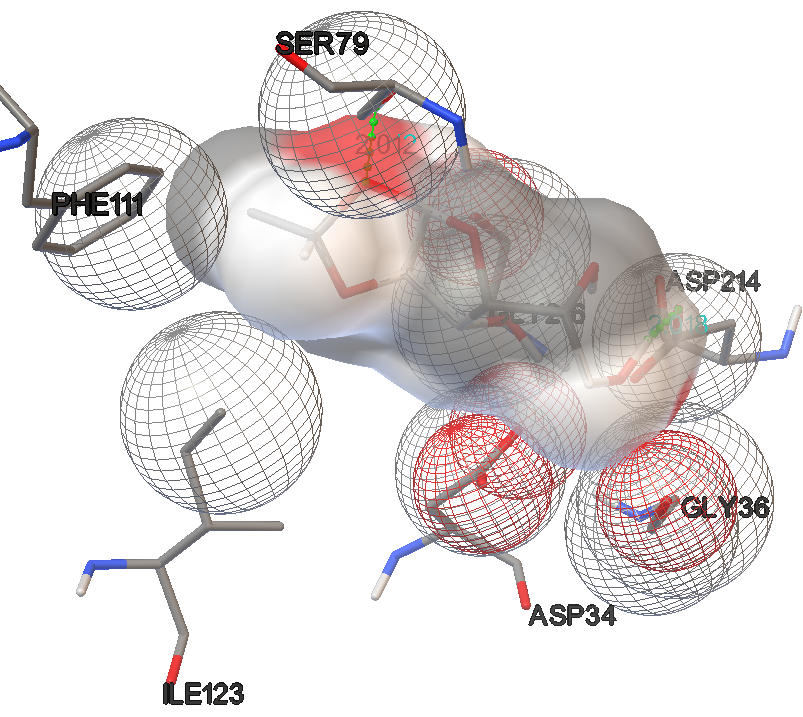
**

**Figure S3.** Molecular docking picture of fragment **3b** (AutoDock).

**
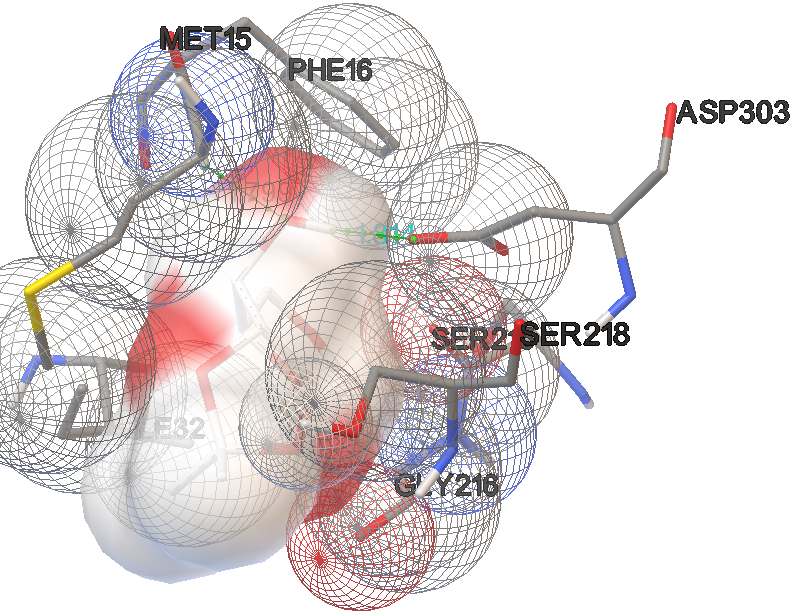
**

**Figure S4.** Molecular docking picture of fragment **3c** (AutoDock).

**
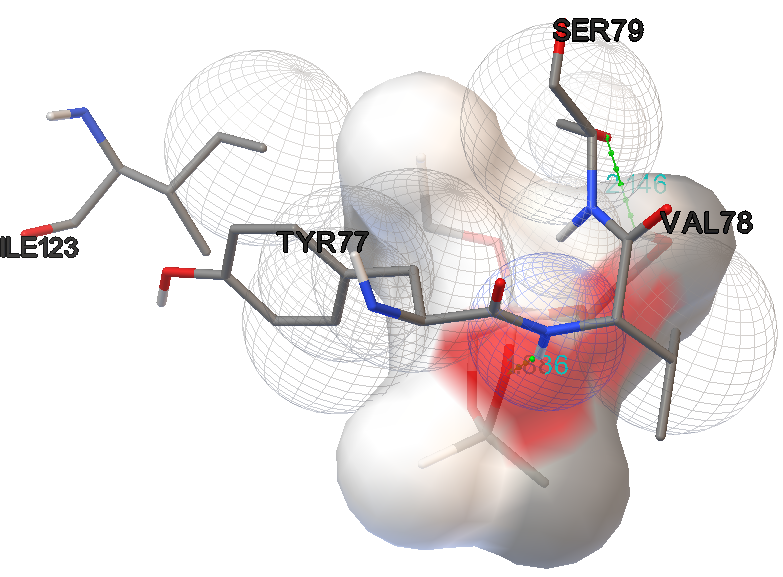
**

**Figure S5.** Molecular docking picture of fragment **3d** (AutoDock).


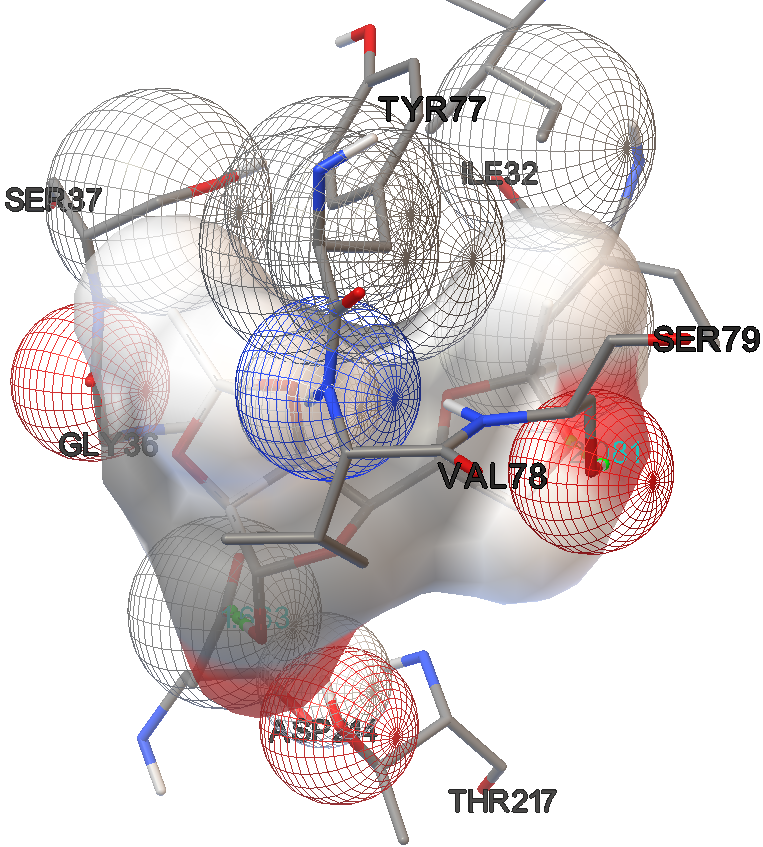


**Figure S6.** Molecular docking picture of fragment **3f** (AutoDock).

**
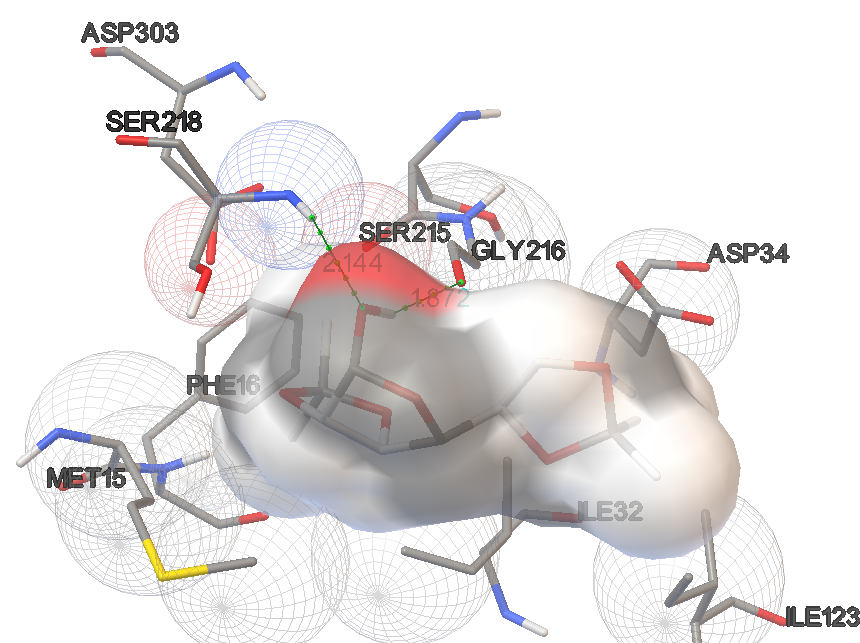
**

**Figure S7.** Molecular docking picture of fragment **3g** (AutoDock).


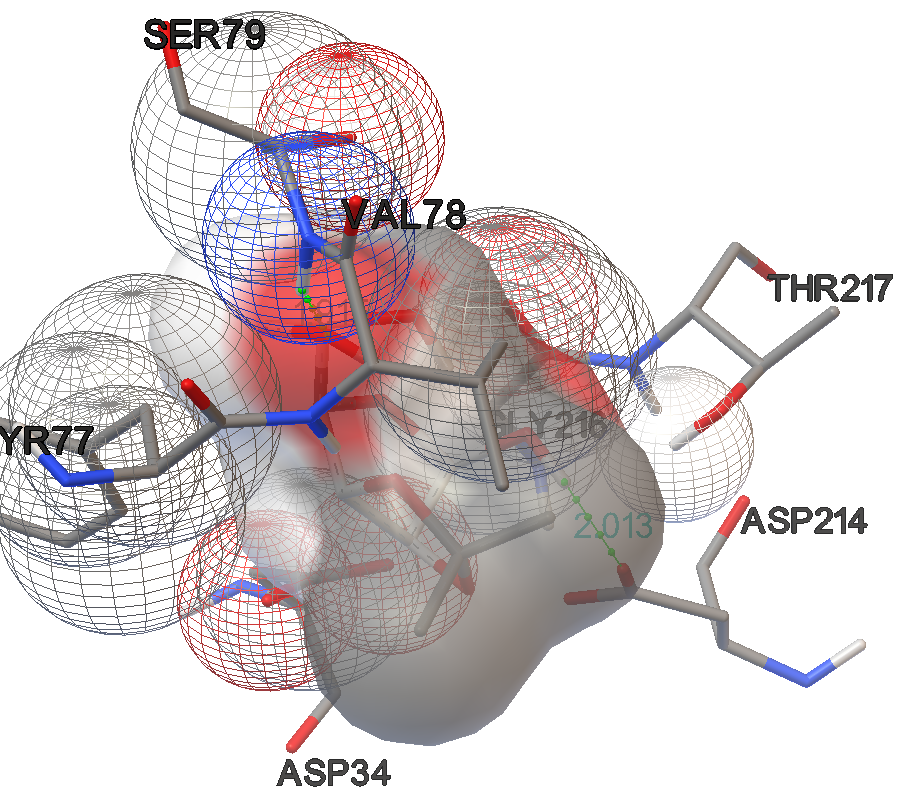


**Figure S8.** Molecular docking picture of fragment **3h** (AutoDock).

**
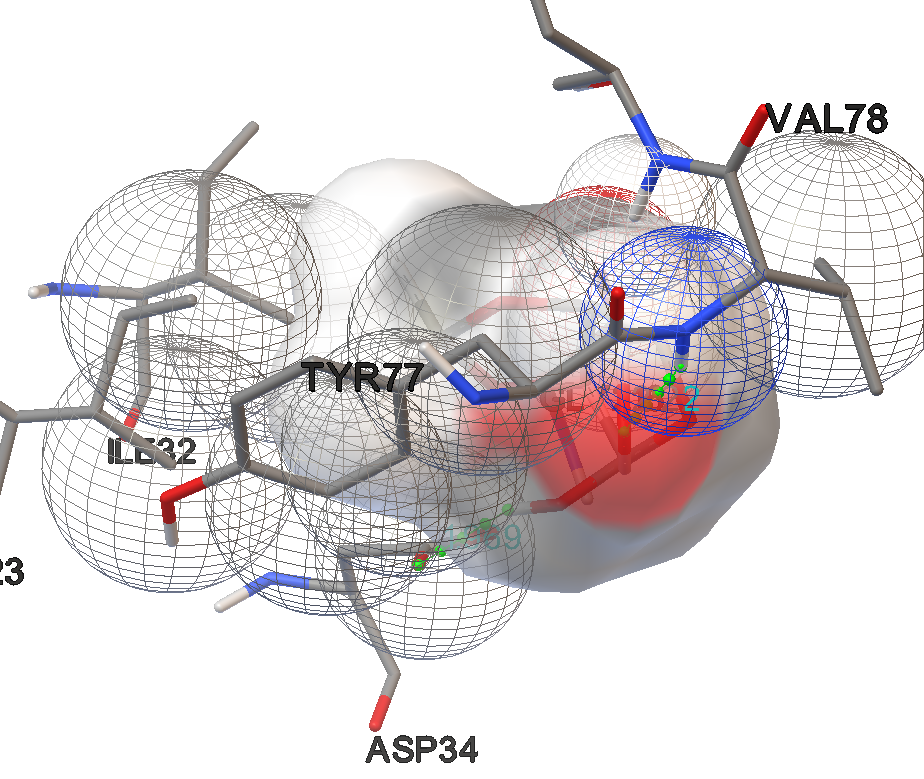
**

**Figure S9.** Molecular docking picture of fragment **3i** (AutoDock).

**
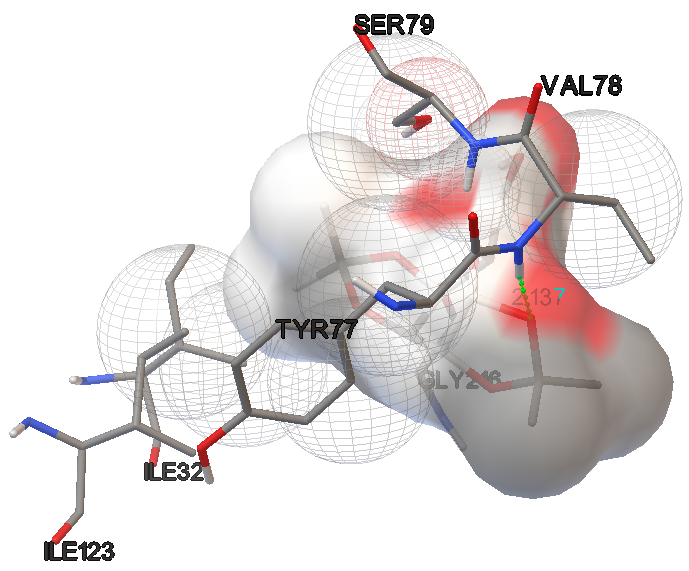
**

**Figure S10.** Molecular docking picture of fragment **3j** (AutoDock).

| **7.** | **Pictures of fragment molecular docking using AutoDock Vina** |
| --- | --- |


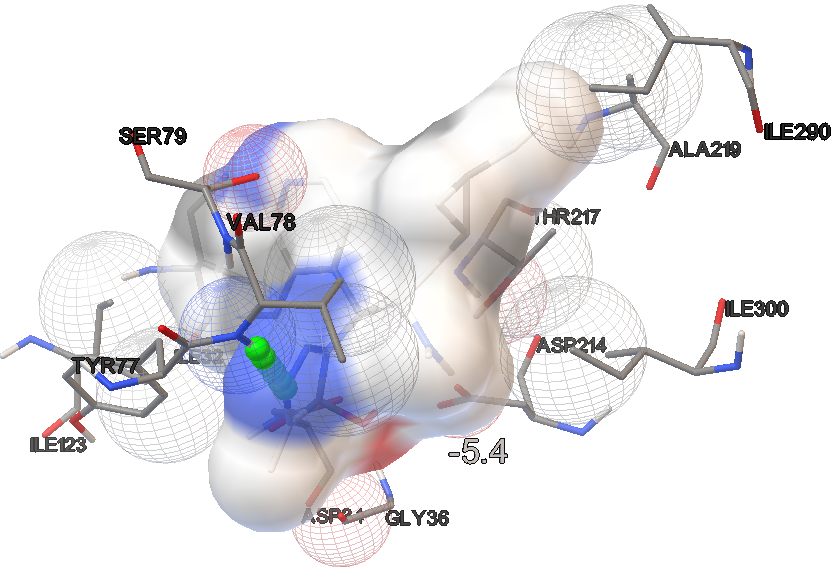


**Figure S11.** Molecular docking picture of central molecule (Cm) fragment (Vina).


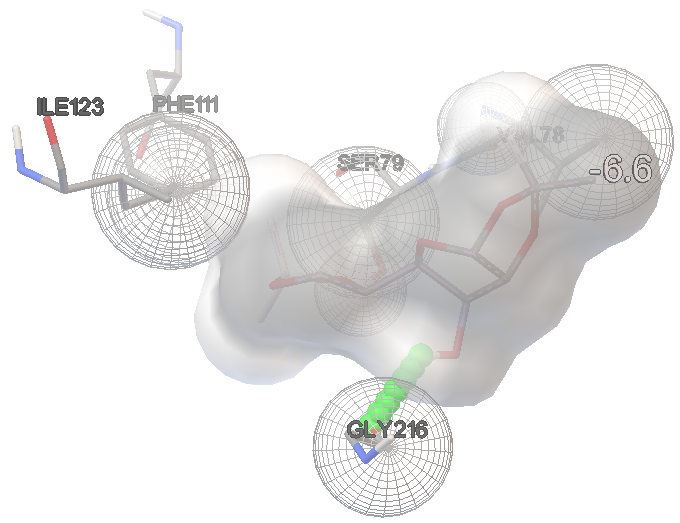


**Figure S12.** Molecular docking picture of fragment **3a** (Vina).

**
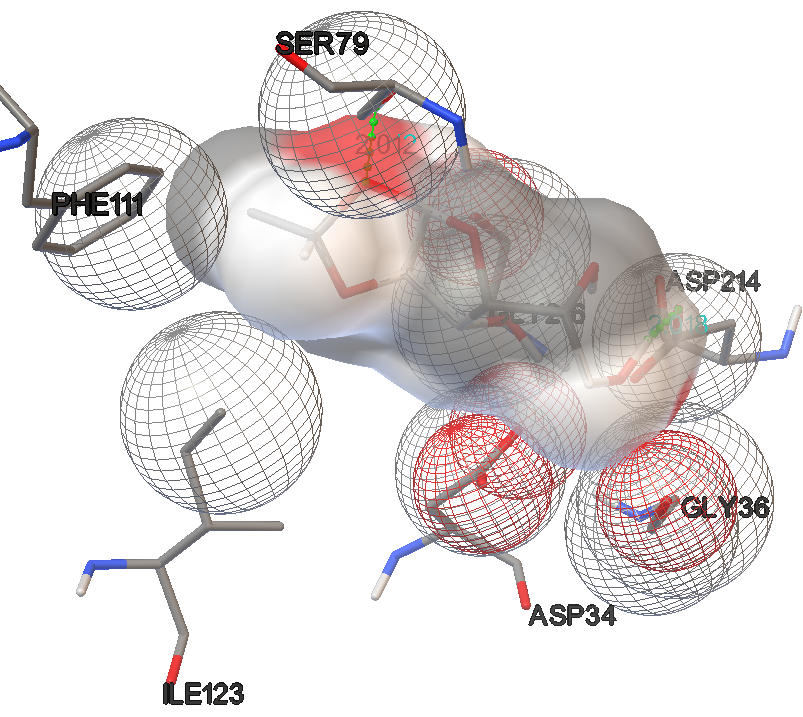
**

**Figure S13.** Molecular docking picture of fragment **3b** (Vina).

**
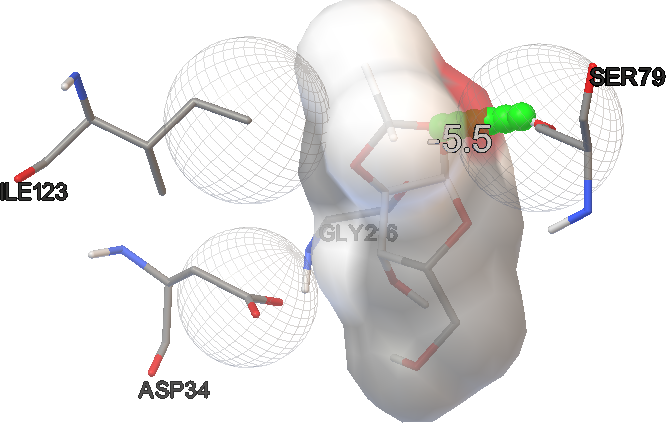
**

**Figure S14.** Molecular docking picture of fragment **3c** (Vina).

**
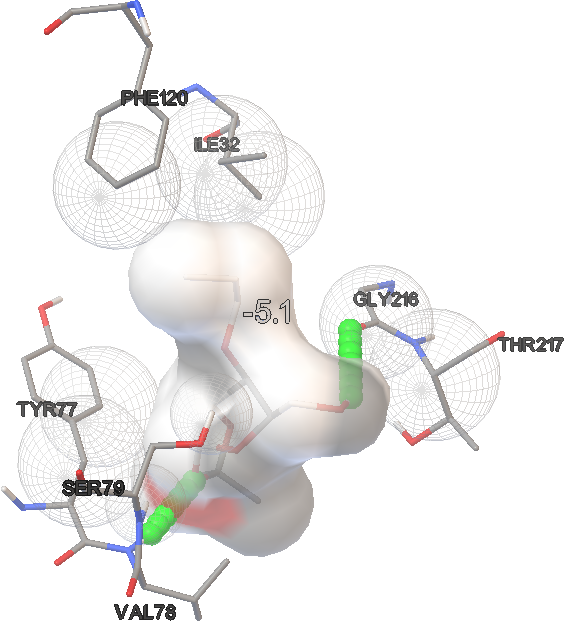
**

**Figure S15.** Molecular docking picture of fragment **3d** (Vina).


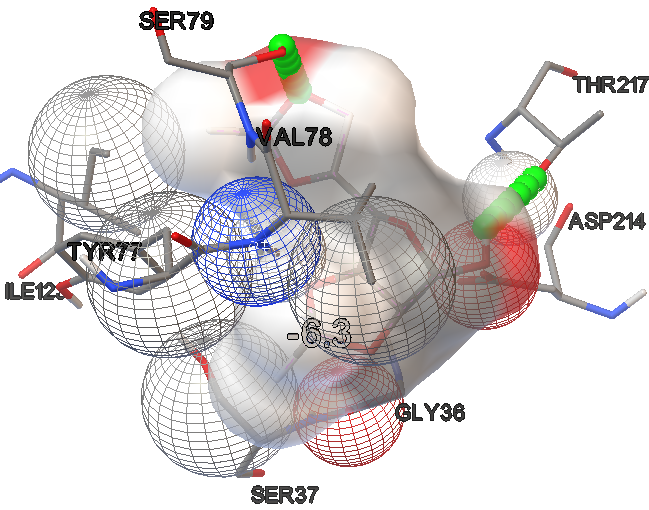


**Figure S16.** Molecular docking picture of fragment **3f** (Vina).

**
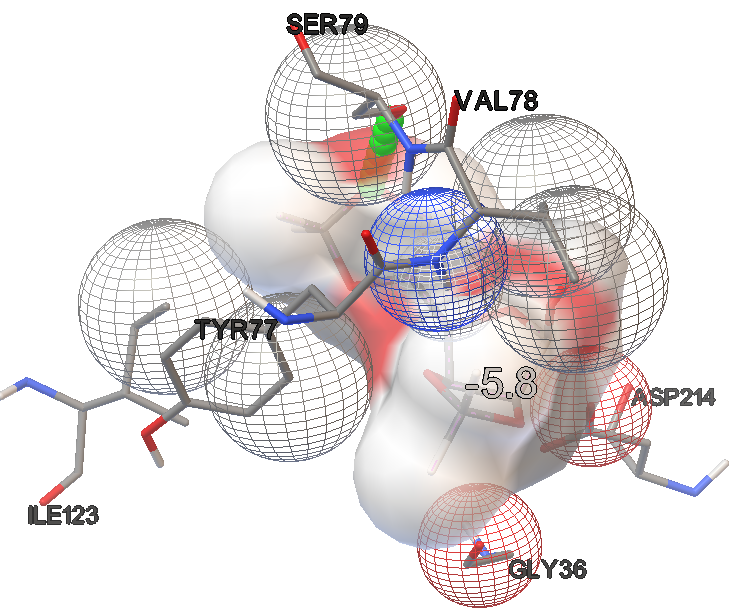
**

**Figure S17.** Molecular docking picture of fragment **3g** (Vina).


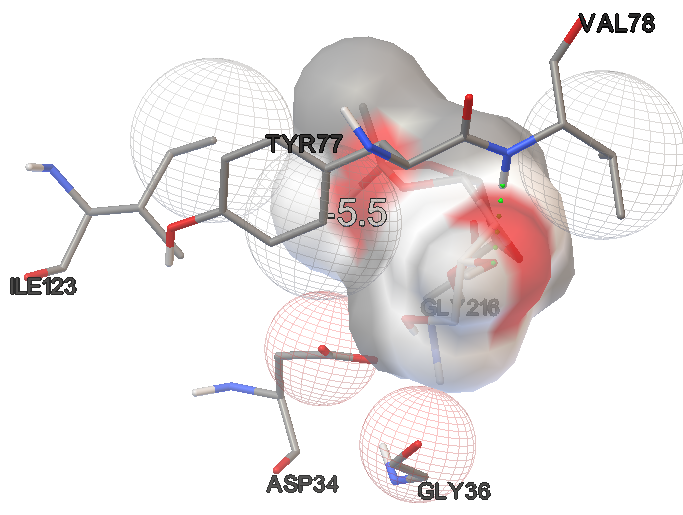


**Figure S18.** Molecular docking picture of fragment **3h** (Vina).

**
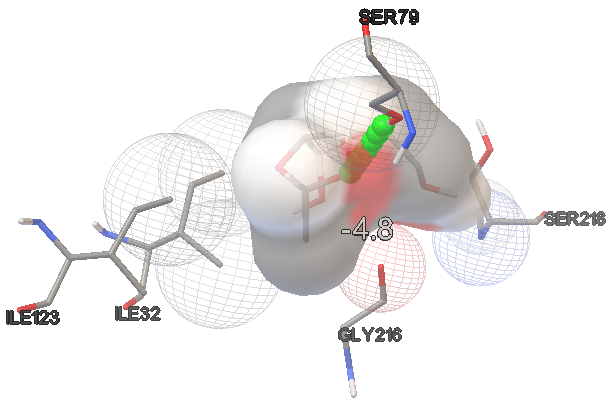
**

**Figure S19.** Molecular docking picture of fragment **3i** (Vina).

**
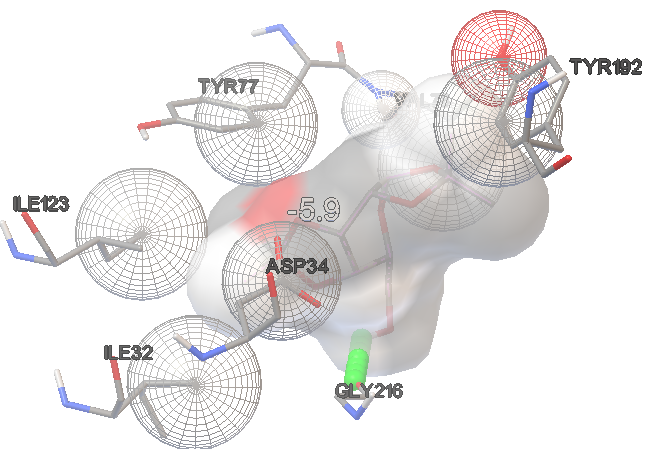
**

**Figure S20.** Molecular docking picture of fragment **3i** (Vina).

| **6.** | **Pictures of Whole molecular docking using AutoDock Vina** |
| --- | --- |


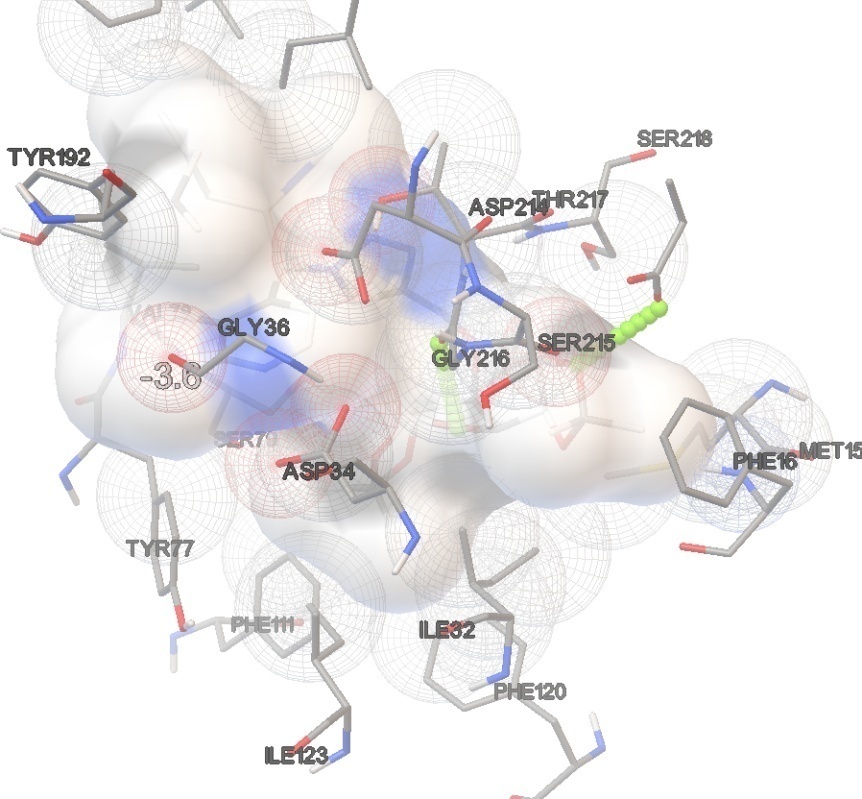


**Figure S21.** Molecular docking picture of whole molecule **3a** (Vina).

**
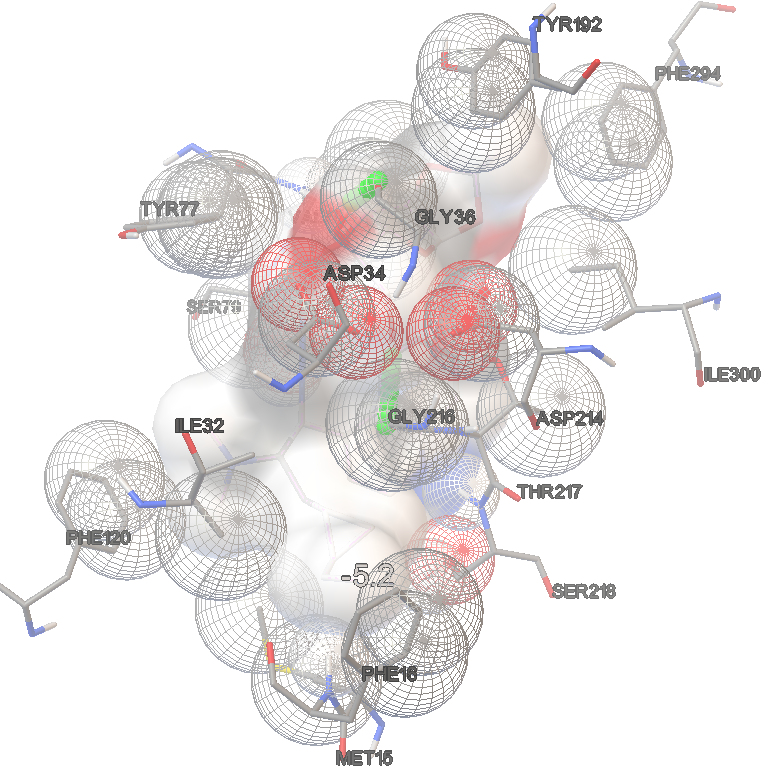
**

**Figure S22.** Molecular docking picture of whole molecule **3b** (Vina).


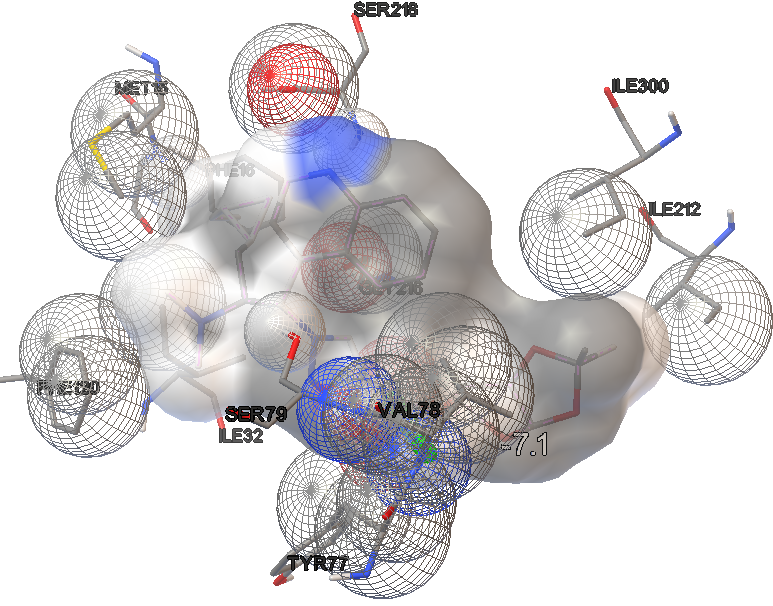


**Figure S23.** Molecular docking picture of whole molecule **3c** (Vina).

**
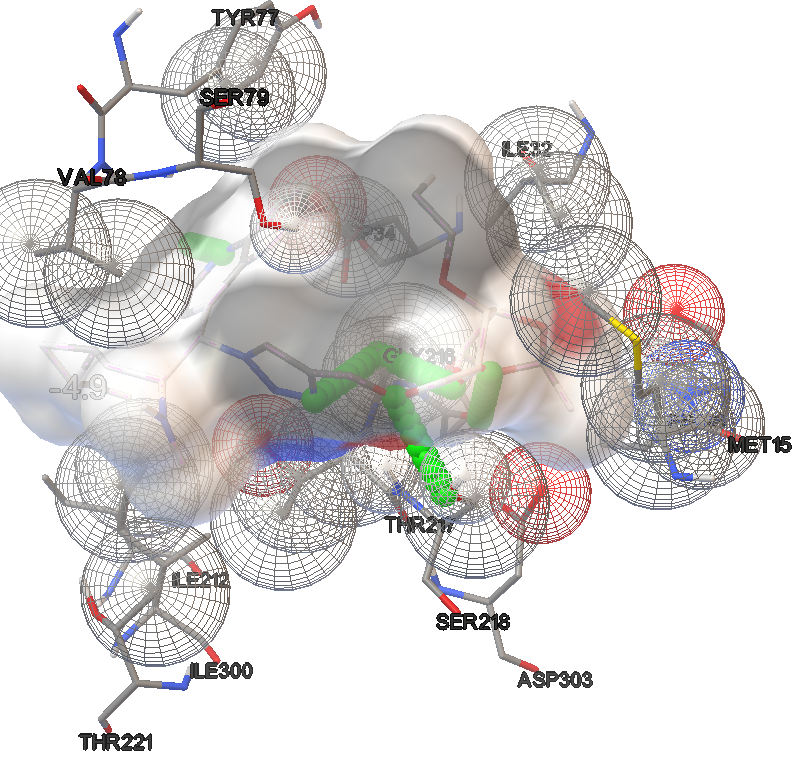
**

**Figure S24.** Molecular docking picture of whole molecule **3d** (Vina).


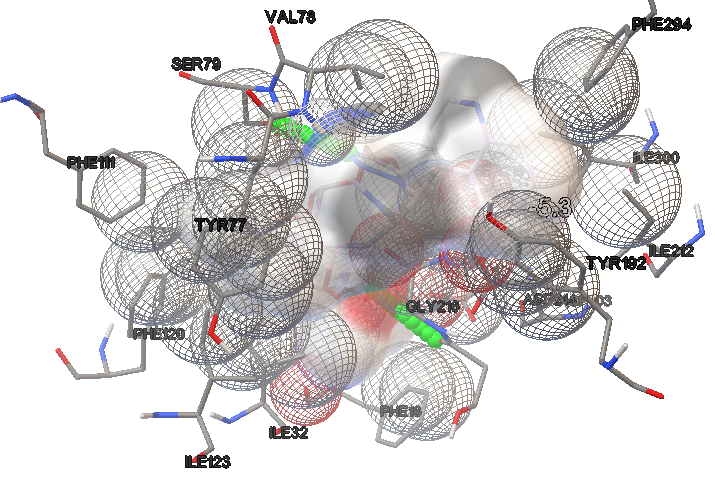


**Figure S25.** Molecular docking picture of whole molecule **3f** (Vina).

**
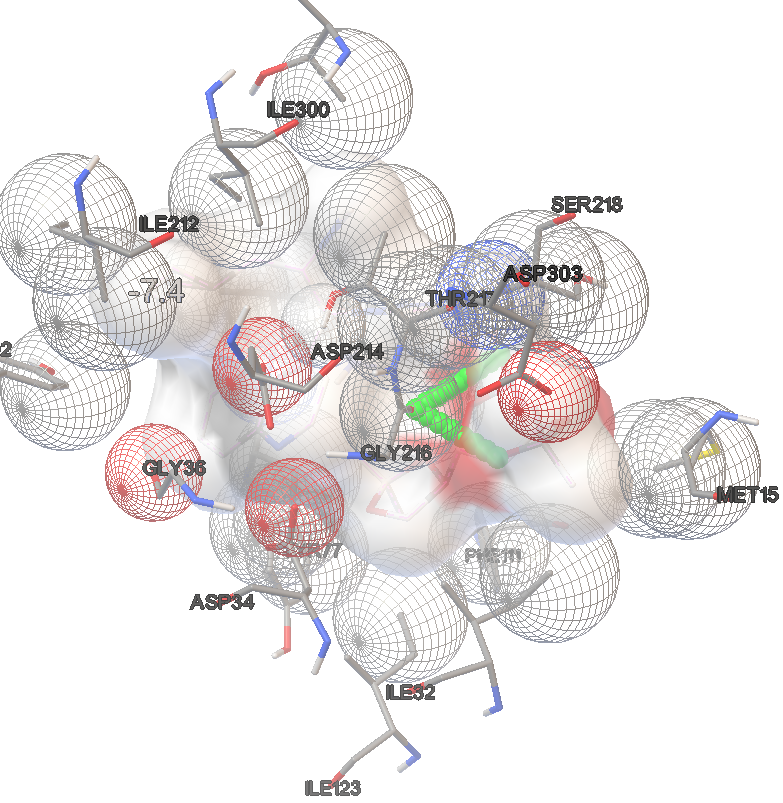
**

**Figure S26.** Molecular docking picture of whole molecule **3g** (Vina).


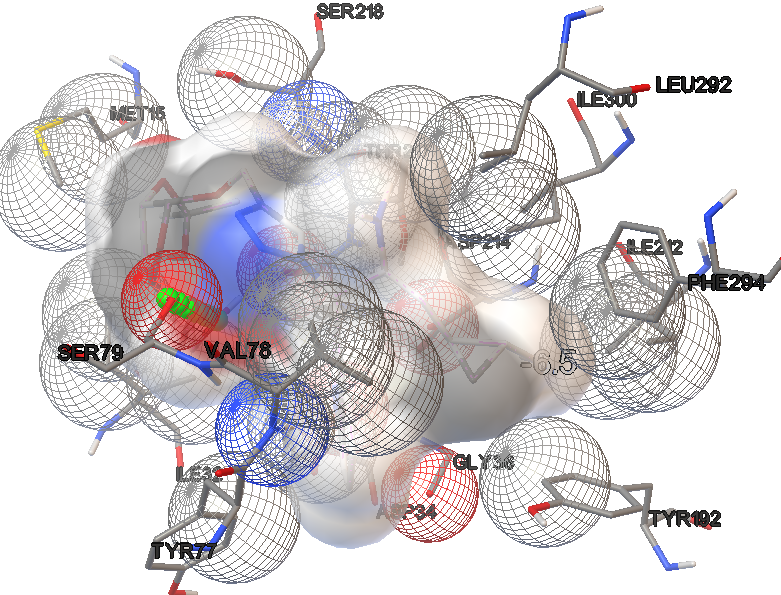


**Figure S27.** Molecular docking picture of whole molecule **3h** (Vina).

**
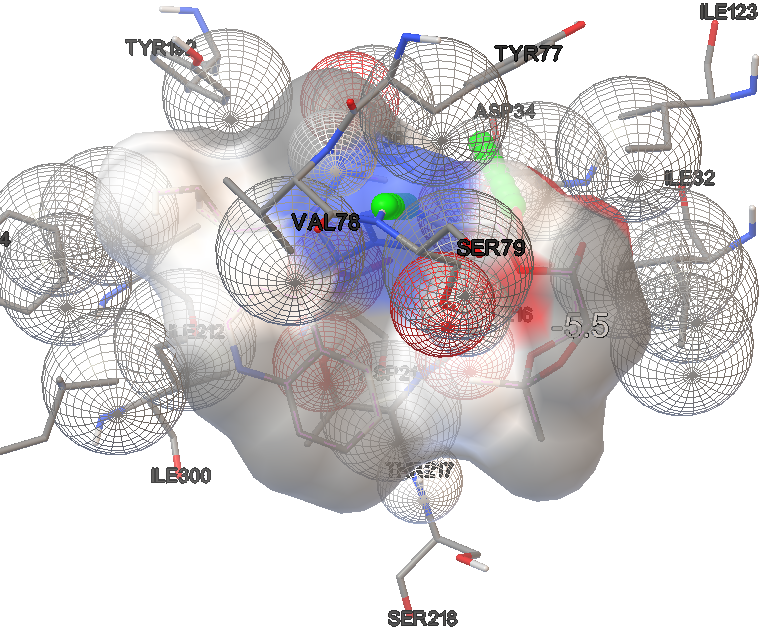
**

**Figure S28.** Molecular docking picture of whole molecule **3i** (Vina).

**
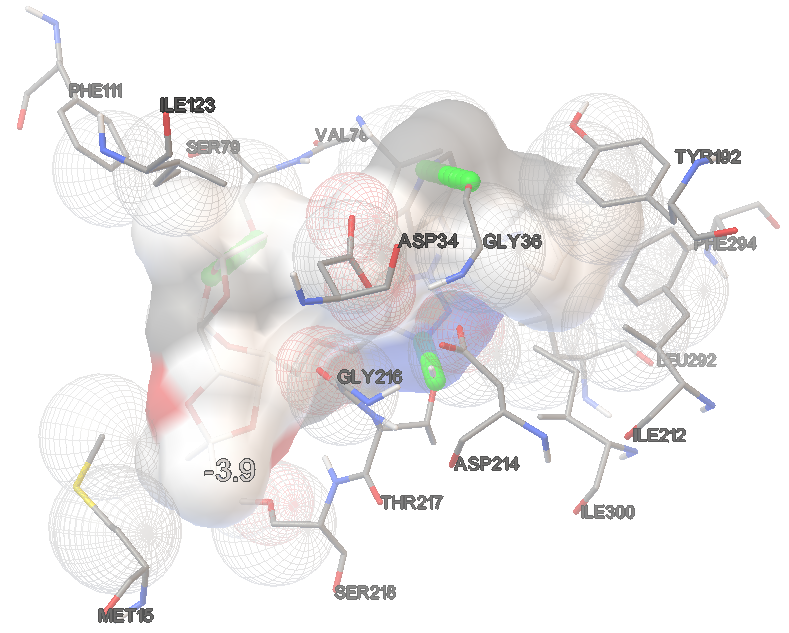
**

**Figure S29.** Molecular docking picture of whole molecule **3j** (Vina).

1. **Table 3.** The characteristic chemical properties computed using optimized structures which directly influences the pharmacokinetics properties of the developed cinchona glycoconjugates

| **Sl No** | **Molecules** | **Molecular weight (AMU)** | **LogP** | | **Molecular Topological Index** | **Charge on molecule** | **VdW**  **(Kcal/mol)** | **Non VdW**  **(kcal/mol)** |
| --- | --- | --- | --- | --- | --- | --- | --- | --- |
|  |  |  | **Molar refractivity** | **Partition coefficient (O/W)** |  |  |  |  |
| 01 | **3a** | 617.749 | 16.5603 | 3.4583 | 53623 | 0 | 45.3653 | -8.398 |
| 02 | **3b** | 577.684 | 15.3463 | 1.2838 | 43911 | 0 | 42.2938 | 0.188 |
| 03 | **3c** | 547.657 | 14.7294 | 2.109 | 38978 | 0 | 92.9424 | 98.4914 |
| 04 | **3d** | 589.738 | 16.1208 | 3.9086 | 50999 | 0 | 97.3504 | 97.1811 |
| 05 | **3e** | 898.12 | 26.1153 | 9.2073 | 161023 | 0 | 69.1903 | -10.3562 |
| 06 | **3f** | 617.749 | 16.5603 | 3.18005 | 54884 | 0 | 96.3809 | 92.9402 |
| 07 | **3g** | 617.749 | 16.5603 | 3.18005 | 54884 | 0 | 176.07 | 117.599 |
| 08 | **3h** | 577.684 | 15.3463 | 1.00555 | 44611 | 0 | 42.5412 | -1.3545 |
| 09 | **3i** | 547.657 | 14.7294 | 1.83075 | 39352 | 0 | 208.086 | 102.528 |
| 10 | **3j** | 617.749 | 16.4773 | 3.3464 | 55973 | 0 | 100.506 | 95.2878 |

The set of molecules has been optimized using gaussian method β3LYP/6-311g(d,p) and optimized structures are computed for the evaluating most proximate value of the chemical properties like molecular weight, molar refractivity, partition coefficient in octanol/water, molar topological index, charge on molecule, Vander Waal force and non Vander Waal force which explains the probable pharmacokinetic behavior of the set. From the values, it is cleared that the set is neither can be termed as completely hydrophobic or hydrophilic rather it is a balanced structure having hydrophobicity and hydrophilicity and the contributing features are logP value, cell interactivity factors and charge stability of the set. Besides, the molecular weight is within 500-650 AMU averagely. This all factors are helpful for the cell permeability as well as cell organelle interactivity in short for good pharmacokinetic features.
